# [^99m^Tc]Tc-PSMA-HSG for PSMA-targeted *H*ybrid *S*urgical *G*uidance: a new addition to the PSMA-I&S/I&T family

**DOI:** 10.1007/s00259-025-07623-2

**Published:** 2025-11-14

**Authors:** Margret Schottelius, David Viertl, Tessa Buckle, Hélène Koch, Sebastian Martin, Alexandra Litvinenko, Marianne Patt, D. M. van Willigen, Fijs W. B. van Leeuwen, Hans-Jürgen Wester, Dorothea Weckermann, Alessandro Liebich, Alexander Gäble, Nic G. Reitsam, Bruno Märkl, Johanna S. Enke, Julia Brosch-Lenz, Constantin Lapa

**Affiliations:** 1 Translational Radiopharmaceutical Sciences, Department of Nuclear Medicine, Department of Oncology, Centre Hospitalier Universitaire Vaudois (CHUV), University of Lausanne, Lausanne, 1011 Switzerland; 2 AGORA, Pôle de recherche sur le cancer, Lausanne, 1011 Switzerland; 3 SCCL Swiss Cancer Center Leman, Lausanne, 1011 Switzerland; 4 Interventional Molecular Imaging Laboratory, Leiden University Medical Center, Leiden, 2333 ZA The Netherlands; 5 Nuclear Medicine, University of Augsburg, 86156 Augsburg, Germany; 6 Chair for Pharmaceutical Radiochemistry, Faculties of Chemistry and Medicine, Technische Universität München, 85748 Garching, Germany; 7 Urology, Medical Faculty, University of Augsburg, 86156 Augsburg, Germany; 8 Pathology, Medical Faculty, University of Augsburg, 86156 Augsburg, Germany; 9 Bavarian Center for Cancer Research, 86156 Augsburg, Augsburg, Germany; 10 Institute of Nuclear Medicine, Glen Burnie, MD USA

**Keywords:** Prostate cancer (PCa), PSMA, Fluorescence guided surgery, Radioguided surgery, Hybrid tracer

## Abstract

**Purpose:**

To develop a dual-labeled PSMA-targeted tracer for radio- and fluorescence-guided surgery (RGS/FGS) with enhanced clinical utility due to optimized pharmacokinetics and tumor targeting.

**Methods:**

Four novel hybrid PSMA ligands with varying cyanine-based fluorophores were comprehensively characterized preclinically. On the basis of its excellent in vitro (logD, plasma protein binding, PSMA-affinity, internalization) and in vivo (stability, clearance kinetics, tumor uptake in LNCaP and PC3-PIp tumor-bearing mice) profile, [^99m^Tc]Tc-PSMA-HSG was selected as the clinical lead compound. Five patients with primary and recurrent prostate cancer underwent [^99m^Tc]Tc-PSMA-HSG SPECT/CT and RGS. Tracer dosimetry was calculated using a MIRDcalc v1.22 protocol.

**Results:**

The PSMA affinity (IC₅₀=38.4 ± 5.3 nM), hydrophilicity (logD =–2.94), and human plasma protein binding of [^99m^Tc]Tc-PSMA-HSG were all nearly identical to those of the non-fluorescent parent compound [^99m^Tc]Tc-PSMA-I&S. Tumor uptake in mice was 11.8 ± 1.5%ID/g at 6 h p.i. (vs. 6.4 ± 1.0%ID/g for [^99m^Tc]Tc-PSMA-I&S). In and ex vivo fluorescence imaging in mice confirmed tumor localization with high signal-to-background ratios. In patients, [^99m^Tc]Tc-PSMA-HSG showed faster clearance and less background uptake than [^99m^Tc]Tc-PSMA-I&S, with notably reduced salivary gland and intestinal accumulation, but a slightly higher whole body effective dose (0.011 ± 0.003 vs. 0.0052 mSv/MBq). Intraoperative gamma detection revealed lymph node metastases in 6/6 tracer-avid lesions, which were confirmed by PSMA-HSG fluorescence microscopy and histopathology. The specificity, selectivity, NPV and PPV of [^99m^Tc]Tc-PSMA-HSG in RGS were 100%, respectively.

**Conclusions:**

The hybrid tracer [^99m^Tc]Tc-PSMA-HSG demonstrates high specificity and favorable pharmacokinetics. Its successful first-in-human application highlights its translational potential for precise intraoperative detection of PSMA-positive lymph node metastases.

**Supplementary Information:**

The online version contains supplementary material available at 10.1007/s00259-025-07623-2.

## Introduction

Pioneering studies on hybrid (nuclear/fluorescent) intraoperative guidance during sentinel lymph node procedures using dual-labeled indocyanine green (ICG)-[^99m^Tc]Tc-nanocolloid [1] have shown how radioguided surgery (RGS) procedures can be strengthened by including fluorescence guidance [2]. This combined surgical guidance has proven particularly valuable during minimally invasive robotic surgery in prostate cancer (PCa) patients [3]. Recently, this approach has been further boosted by the development and progressive maturation of intraoperative imaging modalities such as the da Vinci Xi firefly fluorescence laparoscope [4] and modern DROP-IN Gamma probes [5–7]. Based on the broad availability and ideal gamma emission profile of ^99m^Tc, the clinical application of RGS is currently dominated by this radioisotope [8].

Due to the particularly high expression of prostate specific membrane antigen (PSMA) on more than 90% of primary and recurrent PCs [9], PSMA not only represents a widely exploited target for nuclear diagnostics (PET or SPECT) and theranostics [10], but has also emerged as an interesting surgical target. The successful clinical implementation of PSMA-targeted RGS using e.g. [^111^In]In-PSMA-I&T [11] and [^99m^Tc]Tc-PSMA-I&S ([^99m^Tc]Tc-mas_3_-y-nal-k-Sub-KuE) [12–15] and the proven practical value of the fluorescent component of ICG-[^99m^Tc]Tc-nanocolloid [1] in prostate cancer have triggered various attempts to combine these chemical designs in so-called PSMA-specific “hybrid” tracers. Facilitated by the accessibility of a variety of lead structures with proven clinical utility in PSMA-PET/CT, such as [^68^Ga]Ga-PSMA-11 [16], [^68^Ga]Ga-PSMA-617 [17] and [^68^Ga]Ga-PSMA-I&T [18], various combined PET and fluorescence approaches towards PSMA-specific hybrid tracers have been pursued in recent years [19–24]. These combined efforts have spawned several PSMA-targeted hybrid tracers, including [^68^Ga]Ga-PSMA-I&F [25], a SulfoCy5 containing analogue of [^68^Ga]Ga-PSMA-I&T [18, 26]. Some approaches have even advanced towards the surgical setting using pigs [27] and into first-in-man pilot studies [28–30].

**Fig. 1:**
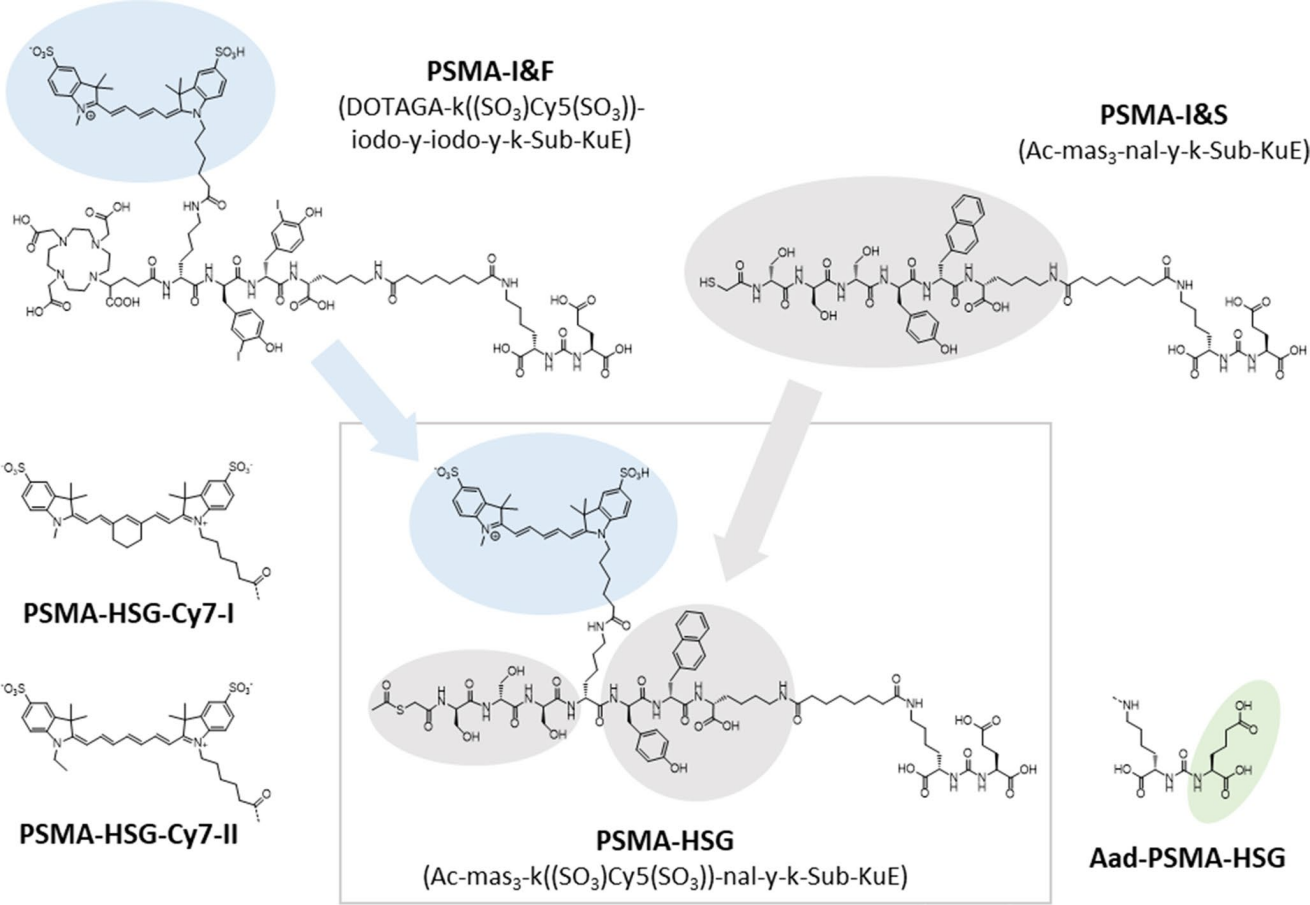
Structures of the PSMA-I&F [25]- and PSMA-I&S [12]-derived novel ligand, PSMA-HSG, and its corresponding Sulfo-Cy7 analogs (left; exchange of the dye component only) and its Aad-derivative (right; Glu-for-Aad exchange in the inhibitor component only)

However, based on the established clinical setup of RGS procedures, there is demand for hybrid PSMA-agents that incorporate ^99m^Tc [31]. The design of [^99m^Tc]Tc-PSMA-I&S with its optimized linker structure [32] has led to its widespread clinical use for PSMA-targeted RGS [13–15, 33–37] and imaging [38, 39]. To exploit its positive features also in the context of Hybrid **S**urgical **G**uidance, [^68^Ga]Ga-PSMA-I&F [25] (Fig. 1) – a hybrid tracer that had allowed effective targeting of PSMA-positive tumors in mice and their visualization via fluorescence imaging – was converted to a second-generation analog based on the PSMA-I&S backbone, termed [^99m^Tc]Tc-PSMA-HSG (Fig. 1).

Of note, due to the particularly high brightness of the far-red dye Sulfo-Cy5, which provides improved tissue penetration compared to near infrared (NIR) dyes such as ICG or Cy7 [40], it was also used as the dye of choice in PSMA-HSG. However, to date, virtually all clinical grade fluorescence laparoscopes are optimized for NIR dyes. Thus, to take this important translational aspect into account, two alternative Sulfo-Cy7-conjugated PSMA-HSG analogs were also included into this study (Fig. 1). Of the Sulfo-Cy7-dyes investigated, one, termed Sulfo-Cy7-I in this study, is commercially available, while the other, Sulfo-Cy7-II is structurally more closely related to commercial Sulfo-Cy5 (in PSMA-HSG) and was thus also investigated to capture potential structure-activity relationships related to the exact dye structure. To be able to assess the general influence of the dye conjugation on overall PSMA-targeting characteristics and in vivo behavior of the different [^99m^Tc]Tc-PSMA-HSG and [^99m^Tc]Tc-PSMA-HSG-Cy7 analogs, they were compared to the parent compound [^99m^Tc]Tc-PSMA-I&S. Since a recently published study had demonstrated that a Glu-for-Aad exchange led to a dramatic reduction in kidney uptake at almost unchanged tumor accumulation of EuK-based PSMA-targeted ligands [41], this modification was also included in the HSG design. Following the head-to-head preclinical comparisons with [^99m^Tc]Tc-PSMA-I&S, the emerging lead compound [^99m^Tc]Tc-PSMA-HSG was evaluated in a first-in-human study.

## Materials and methods

### Precursor synthesis

The respective PSMA-HSG- and Aad-PSMA-HSG precursor backbones were synthesized using a novel, optimized solid-phase synthesis procedure. Only the final step, the conjugation with the respective fluorescent dye (Sulfo-Cy5, Sulfo-Cy7-I or Sulfo-Cy7-II) using COMU as the coupling reagent, was performed in solution phase. Detailed synthesis protocols and ligand characterizations via HPLC and MS (Supplementary Fig. 1) are provided in the Supplementary Material.

### Photophysical characterization

The determination of the quantum yield and molar extinction coefficient of PSMA-HSG were performed according to previously published procedures [42].

### Radiolabeling

Labeling with ^99m^Tc was carried out using reaction vials containing a lyophilized mixture of the precursor, buffer and SnCl_2_ as reducing agent. The exact composition of the reaction mixture is provided in the Supplementary Material. When high specific activities were required (in vitro studies, biodistribution at 0.1 nmol peptide dose), reaction vials containing 5 or 10 nmol of labeling precursor were used. For SPECT/CT imaging and biodistribution studies at 1 nmol peptide dose as well as for patient application, vials containing 25, 120 of 167 nmol of labeling precursor were used. SPE purification and subsequent reconstitution in PBS provided [^99m^Tc]Tc-PSMA-HSG and the reference [^99m^Tc]PSMA-I&S in ≥ 98% radiochemical purity (as determined by radio-TLC and radio-HPLC).

### Determination of lipophilicity

The lipophilicity of [^99m^Tc]Tc-PSMA-HSG was determined via a modified shake-flask method as described [43].

### In vitro evaluation

Competitive binding experiments (IC_50_) were carried out in accordance with an established protocol [43] using PSMA-expressing LNCaP cells and ([^125^I]I-BA)KuE as standard radioligand. Internalization kinetics of [^99m^Tc]Tc-PSMA-HSG (0.5 nM) were investigated in a dual tracer internalization assay using PSMA expressing LNCaP cells and ([^125^I]I-BA)KuE (0.1 nM) as an internal reference. Non-specific binding/internalization was determined in the presence of 10 µM 2-PMPA. Furthermore, PSMA-HSG internalization into LNCaP cells was investigated using fluorescence microscopy (100 nM PSMA-HSG). Please see the Supplementary Material for experimental details.

### Plasma protein binding (B_50_)

Half-maximal binding (B_50_) of [^99m^Tc]Tc-PSMA-HSG, [^99m^Tc]Tc-PSMA-I&S and [^177^Lu]Lu-PSMA-I&T to human serum albumin (HSA), human serum and mouse serum was quantified using an ultrafiltration method (MW cutoff 30 kDa) based on a previously published protocol with minor modifications [44]. A detailed experimental procedure is provided in the Supplementary Material.

### In vivo evaluation of [^99m^Tc]Tc-PSMA-HSG

All animal experiments were conducted in compliance with the Swiss legislation for care and use of laboratory animals under the license VD-3595.

### In vivo stability study

The in vivo stability of [^99m^Tc]Tc-PSMA-HSG at 1 h p.i. was investigated via radio-RP-HPLC analysis of blood and urine samples. Due to its poor extraction efficiency from liver, the hepatic metabolism of [^99m^Tc]Tc-PSMA-HSG by liver enzymes was investigated using a mouse liver homogenate (30 min incubation at 37 °C). Detailed experimental procedures are provided in the Supplementary Material.

### Biodistribution studies

The biodistribution of [^99m^Tc]Tc-PSMA-HSG and of the reference compound [^99m^Tc]Tc-PSMA-I&S was investigated in LNCaP xenograft bearing NSG mice (see supplemental information).

Mice were injected intravenously with the respective ^99m^Tc-labeled tracer (24–34 MBq (fixed peptide dose of 1 nmol/mouse) or 3.7–7.6 MBq (fixed peptide doses of 0.1 nmol/mouse), respectively) and were sacrificed at 2 h (1 nmol-cohort) or 6 h p.i. (0.1 nmol-cohort), respectively. The organs of interest were dissected, and the activity concentration in weighed tissue samples was quantified using an AMG Automatic Gamma Counter (Hidex, Turku, Finland). Biodistribution data are given in %iD/g and represent means ± SD (groups of *n* = 5 animals). Statistical analysis (Multiple unpaired t-test) of biodistribution data and tumor/organ ratios was performed using GraphPad Prism (Boston, USA).

### Small animal SPECT/CT imaging

SPECT/CT images were acquired using an Albira Si PET/SPECT/CT (Bruker Biospin Corporation, Woodbridge, CT, USA) instrument. Mice were injected with the respective ^99m^Tc-labeled tracer (24–34 MBq; the injected peptide amount was kept constant at 1 nmol/animal), with (blocking) or without (control) coinjection of 226 µg (1 µmol) 2-PMPA. Mice were allowed to stay awake for 2 h and were then anesthetized for the duration of the imaging experiments by inhalation of 1.5% isoflurane/O_2_ and placed on a heated bed (30–35 °C).

For SPECT imaging, static acquisitions of 2 h (accumulation of 1–2 × 10^6^ events) were acquired with the following parameters: photopeak at 140 keV ± 30%, axial FOV 82.5 mm using a single pinhole collimator. For reconstruction, an ordered subset expectation-maximization algorithm (OSEM) with two iterations was used, and scatter correction was applied. For CT, the following parameters were used: 400 µA intensity and 35 kV voltage, 600 projections. Images were reconstructed using a filtered back-projection (FBP) algorithm with de-ringing correction. Fused representative SPECT/CT images were acquired using the PMOD software (PMOD technologies, version 3.709, Zurich, Switzerland).

### Fluorescence imaging in mice

Fluorescence imaging of PC3-Pip xenografts in NSG mice (2 h p.i. at a PSMA-HSG dose of 1 nmol and 6 h p.i. at a ligand dose of 0.1 nmol, respectively; *n* = 1 per compound and condition) was performed using a clinical-grade fluorescence laparoscopy set-up (KARL STORZ Endoskope GMBh & Co. KG). Cy5-based tumor imaging was performed using D-light P system and a prototype modified IMAGE 1 S light source with integrated Cy5 filter (both KARL STORZ Endoskope GMBh & Co. KG). Images were recorded using integrated KARL STORZ software and image processing was performed using custom MATLAB software that provided a signal intensity-based representation of the signal-to-background ratio (SBR; [45, 46]).

### Confocal fluorescence imaging of excised tumor specimens

After in vivo imaging, the tumors (*n* = 1 per condition) were excised and cut into 2 mm slices. Prior to imaging, slices were placed on a 35-mm culture dish containing a glass insert (MatTek Co.). Fluorescence confocal imaging was then performed using a Leica SP8 WLL microscope (Leica Microsystems). For assessment of tracer uptake Cy5 settings were used; λ_ex_ = 633 nm, λ_em_ = 650–700 nm. Images were processed using accompanying LASX software (Leica Application Software Suite 4.8).

### Clinical SPECT/CT imaging and dosimetry

Five patients with a diagnosis of initial (*n* = 4) or biochemical recurrence of (*n* = 1) histologically proven prostate cancer and suspicion of lymph node metastases as determined by prior PSMA-directed PET imaging underwent [^99m^Tc]Tc-PSMA-HSG imaging at the Department of Nuclear Medicine of the University Hospital Augsburg, Germany. For all patients, radioguided surgery had been recommended by the local multidisciplinary tumor board. The administration of [^99m^Tc]Tc-PSMA-HSG (doses of 53 µg (*n* = 3), 250 µg (*n* = 1) and 350 µg (*n* = 1), respectively) complied with the German Medicinal Products Act, Arzneimittelgesetz § 13.2b, and the responsible regulatory bodies. The lowest dose (53 µg, 25 nmol) corresponds to the standard clinical dose used in [^99m^Tc]Tc-PSMA-I&S imaging and RGS. The more elevated doses were specifically selected to match doses that had previously been shown to provide sufficient fluorescent signal for in vivo/ex vivo fluorescence detection [47] in a large animal model.

All patients gave written informed consent prior to injection of the radiotracer. This retrospective evaluation was approved by the local ethics committee of the Ludwig-Maximilians-University Munich (reference number: 25–0493). Safety was assessed by monitoring adverse events up to 24 h after administration of [^99m^Tc]Tc-PSMA-HSG.

### Imaging and dosimetry protocol

[^99m^Tc]Tc-PSMA-HSG scans were performed using a GE Discovery NM/CT 670 Pro (GE Healthcare, Milwaukee, USA; *n* = 4) or Siemens Symbia T2 (Siemens Healthineers, Erlangen, Germany; *n* = 1) system. For dosimetry, planar dynamic whole body scans were performed at 5 min and 1 h after injection with a bed speed of 30 cm/min; and at 3–4 h and 20 h after injection with a speed of 12 cm/min. At 3–4 h and 20 h after injection additional SPECT/CT imaging was acquired. Attenuation maps were generated on the basis of low-dose CT. For a detailed protocol of imaging parameters and the dosimetry protocol please refer to the Supplementary Material.

### Radioguided surgery

Radioguided open surgery was performed the day after tracer injection by an experienced urologic surgeon (DW; more than 20 years of surgical experience) in collaboration with a nuclear medicine physician. Using a conventional gamma probe (C-Trak^®^, SanuC GmbH, Jahnsdorf, Germany), suspicious lymph nodes (defined as spots with signal-to-background ratios ≥ 2) were excised separately followed by pelvic lymph node dissection. All excised lesions were re-examined ex vivo prior to further histopathological work-up. Fresh frozen tissue sections were prepared using a standard cryosectioning protocol with 10–20 μm sections mounted for fluorescence imaging. These slides were stored on dry ice prior to fluorescence microscopy to preserve tissue integrity. Fluorescence microscopy of patient samples was performed using an Axioscan 7 slide scanner (Zeiss) equipped with a Colibri 7 LED light source and a 20×/0.8 NA Plan-Apochromat objective. Cy5 fluorescence was acquired using a filter set with excitation 640/30 nm, dichroic mirror 660 nm, and emission 690/50 nm. Image acquisition and analysis were performed using ZEN Blue 3.5 software (Zeiss).

For immunohistochemical analysis, adjacent formalin-fixed, paraffin-embedded tissue sections were cut at 2 μm thickness and stained for PSMA (clone EP192 BioSB, BIOO Scientific) using a Ventana Benchmark automated platform (Bio SB, Santa Barbara, CA, USA). Conventional hematoxylin and eosin (H&E) and immunohistochemical (for PSMA) staining was performed on corresponding tissue sections to enable morphologic correlation and verification of target expression, respectively. For further information on immunhistochemical staining please refer to the Supplementary Material.

## Results

### Synthesis

As opposed to PSMA-I&F and PSMA-I&S, which were both synthesized using an established fragment condensation strategy [12, 25], the entire PSMA-HSG backbone was assembled step-wise on solid support (see supplementary material for a detailed synthesis scheme). This change in synthesis protocol was necessary because both the 2-mercaptoacetyl group in mas_3_ (even in the acetyl protected form) and Sulfo-Cy5/7, respectively, are unstable under the reaction conditions required for the respective orthogonal amino protection/deprotection steps during SPPS.

By using the novel solid phase approach based on commercially available Fmoc-Glu(OtBu)-Wang resin and the exclusive use of acid labile side chain protecting groups, however, the fully deprotected Ac-mas_3_-k-y-nal-k-Sub-KuE backbone was obtained in reasonable yield (30% crude product based on resin-bound Fmoc-Glu(OtBu)-OH). Lower yields were obtained for the 2-CTC-resin-based SPPS of Ac-mas_3_-k-y-nal-k-Sub-Ku-Aad (8%), most probably due to inefficient resin loading with the first amino acid. Only the last synthesis step, the direct conjugation of the preactivated Sulfo-Cy5/Cy7 carboxylic acid to the free D-Lys-sidechain, was carried out in solution (as previously reported for PSMA-I&F [25]). Upon semipreparative HPLC purification, PSMA-HSG and its respective Cy7-I, Cy7-II and Aad-analogs were obtained in 90–98% purity and 9–21% yield based on starting peptide. As opposed to PSMA-I&S [12], the acetyl protecting group of the mas_3_ chelator was maintained in the final product, since it is cleaved under the reducing conditions during the ^99m^Tc-labeling reaction and prevents unwanted thiol oxidation during storage of the precursor.

### In vitro evaluation

The in vitro PSMA targeting characteristics of [^99m^Tc]Tc-PSMA-HSG and its analogs are summarized in Table 1. For comparison, data for the corresponding reference compounds, i.e. [^125^I]IBA-KuE, [^99m^Tc]Tc-PSMA-I&S, and [^68^Ga]Ga-PSMA-I&F, all of which had been assayed under identical experimental conditions, were also included.

**Table 1:** PSMA affinities and internalization (in % of the internal reference ([^125^ I]IBA)KuE after 60 min incubation at 37 °C, LNCaP cells) of the novel hybrid ligands and selected reference compounds [12, 25]

Ligand	IC_50_ [nM]	Tracer	internalization [% of reference]*
(IBA)KuE	7.1 ± 2.4	([^125^I]IBA)KuE	100
PSMA-I&S^#^	39.7 ± 1.2	[^99m^Tc]Tc-PSMA-I&S	103 ± 10
[^nat^Ga]PSMA-I&F	10.5 ± 2.1	[^68^Ga]Ga-PSMA-I&F	103 ± 9
PSMA-HSG^#^	33.8 ± 8.7	[^99m^Tc]Tc-PSMA-HSG	14 ± 1
PSMA-HSG-Cy7-I ^**#**^	70.7 ± 10.8	[^99m^Tc]Tc-PSMA-HSG-Cy7-I	17 ± 2
PSMA-HSG-Cy7-II ^**#**^	73.1 ± 11.1	[^99m^Tc]Tc-PSMA-HSG-Cy7-II	28 ± 7
Aad-PSMA-HSG ^**#**^	512 ± 31	[^99m^Tc]Tc-Aad-PSMA-HSG	n.d.

* The specific internalization (total internalized activity corrected by internalization in the presence of 10 µM 2-PMPA) of the respective tracer of interest and of the reference compound ([^125^I]I-BA)KuE was determined in a dual trace experiment. The internalization of ([^125^I]I-BA)KuE was used for data normalization. Data show relative internalization after 1 h at 37 °C.

^**#**^ free mas_3_-chelator.

With an IC_50_ of 33.8 ± 8.7 nmol, PSMA-HSG shows a slightly improved PSMA-affinity compared to the parent compound PSMA-I&S [12]. Conversely, conjugation with either of the two SulfoCy7 analogs investigated led to a twofold decreased PSMA affinity of PSMA-HSG-Cy7-I and -Cy7-II compared to PSMA-HSG, respectively, independently of the exact dye structure. In contrast to reports from the literature [41], the Aad-for-Glu substitution in the inhibitor component of PSMA-HSG was not well tolerated, leading to a fifteen-fold reduction in PSMA-affinity for Aad-PSMA-HSG. The compound was therefore excluded from further evaluation.

**Fig. 2:**
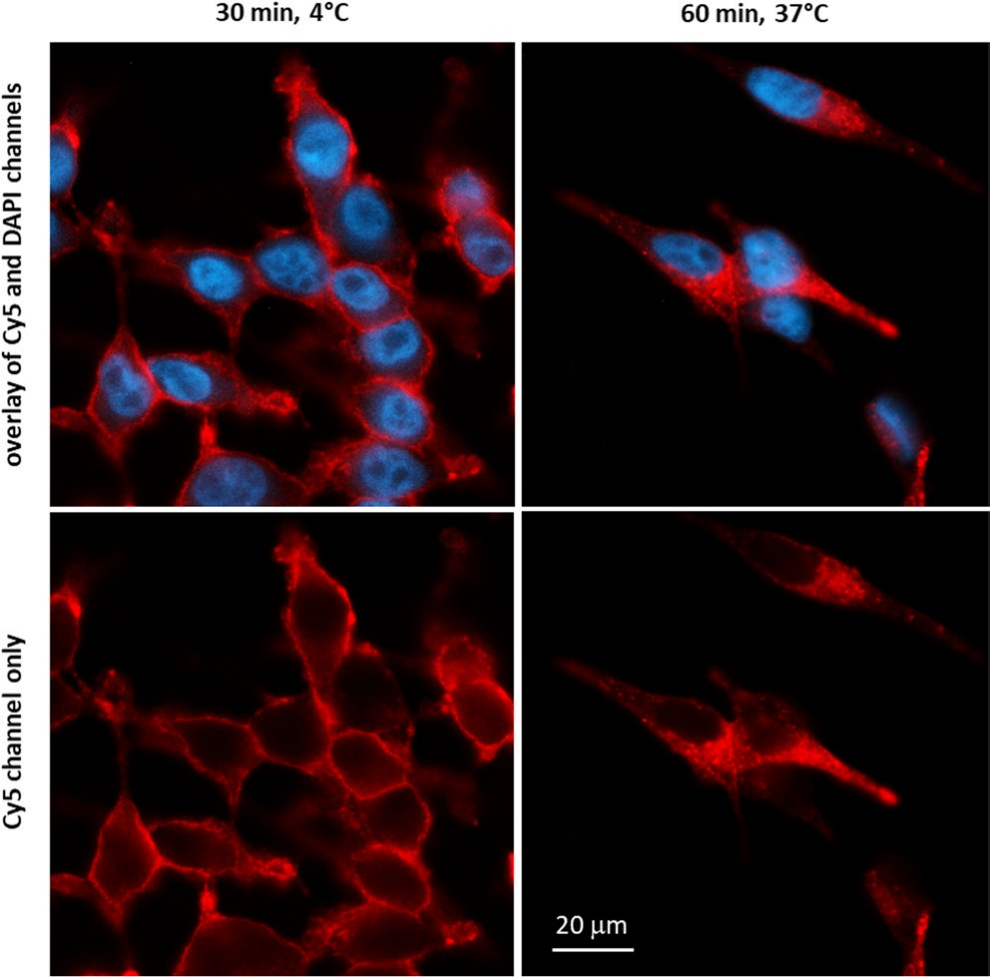
Fluorescence microscopy (63-fold magnification) of LNCaP cells after incubation with 100 nM PSMA-HSG for 30 min at 4 °C (membrane binding, left column) and for 60 min at 37 °C (internalization, right column row). Cells were fixed with 4% paraformaldehyde, and nuclei were stained with 300 nM DAPI

Dual tracer internalization studies revealed a consistently reduced internalization for all [^99m^Tc]Tc-PSMA-HSG analogs compared to [^99m^Tc]Tc-PSMA-I&S and [^68^Ga]Ga-PSMA-I&F, independently of receptor affinity. Nevertheless, for all three compounds, nearly the entire cellular activity was internalized at the time points investigated (10, 30, 60 min), with very low levels of membrane bound (i.e. acid releasable) ligand (Supplementary Fig. 2). To visually confirm sufficient ligand internalization for the intended purpose, the internalization of PSMA-HSG into LNCaP was also assessed using fluorescence microscopy (Fig. 2). While incubation at 4 °C (preventing internalization) revealed intense and PSMA-specific membrane staining (also see Supplementary Fig. 3), incubation at 37 °C led to efficient ligand translocation into the cells.

### Lipophilicity and plasma protein binding

Since the lipophilicity and plasma protein binding of targeted tracers strongly influences their pharmacokinetics, excretion route and target accumulation [48], both were also determined for the novel hybrid analogs in this study and compared to the data obtained for selected reference compounds (Table 2). Interestingly, introduction of SulfoCy5 did not significantly increase the lipophilicity of [^99m^Tc]Tc-PSMA-HSG compared to the parent compound [^99m^Tc]Tc-PSMA-I&S. In line with literature reports [40], however, extending the conjugated indocyanine dye system to Cy7 led to an increased lipophilicity. This effect was, not unexpectedly, slightly more pronounced for the commercial SulfoCy7-analog (Cy7-I) with the central cyclohexene moiety (Fig. 1).

**Table 2:** Lipophilicities (LogD) and tracer affinity (B_50_) to plasma proteins in human serum (HS), mouse serum (MS) and a solution of human serum albumin (HSA) in PBS. B_50_ values are unitless and represent the molar excess of protein over radioligand needed to achieve half-maximal binding of the novel hybrid ligands and selected reference compounds [12, 25, 32] (mean ± SD, n = 3)

Tracer	lipophilicity [logD]	B_50_ HS	B_50_ HSA	B_50_ MS
[^177^Lu]Lu-PSMA-I&T ^#^	−4.10	923 ± 116	1159 ± 46	734 ± 308
[^99m^Tc]Tc-PSMA-I&S	−3.01	321 ± 18	324 ± 6	652 ± 77
[^68^Ga]Ga-PSMA-I&F	−3.40	-	-	-
[^99m^Tc]Tc-PSMA-HSG	−2.94	220 ± 35	273 ± 36	142 ± 22
[^99m^Tc]Tc-PSMA-HSG-Cy7-I	−2.63	246 ± 27	165 ± 21	93 ± 16
[^99m^Tc]Tc-PSMA-HSG-Cy7-II	−2.71	121 ± 16	149 ± 14	53 ± 7

^**#**^ LogD value cited from [32]

Since values for absolute plasma protein binding (in % of added activity) are highly dependent on the experimental setup and on which activity values are used for calculation (e.g. including or excluding filter-bound activity in the case of ultrafiltration methods), an alternative method to determine half-maximal ligand binding (B_50_) and thus their relative affinity to plasma proteins was used [44, 49].

**Fig. 3:**
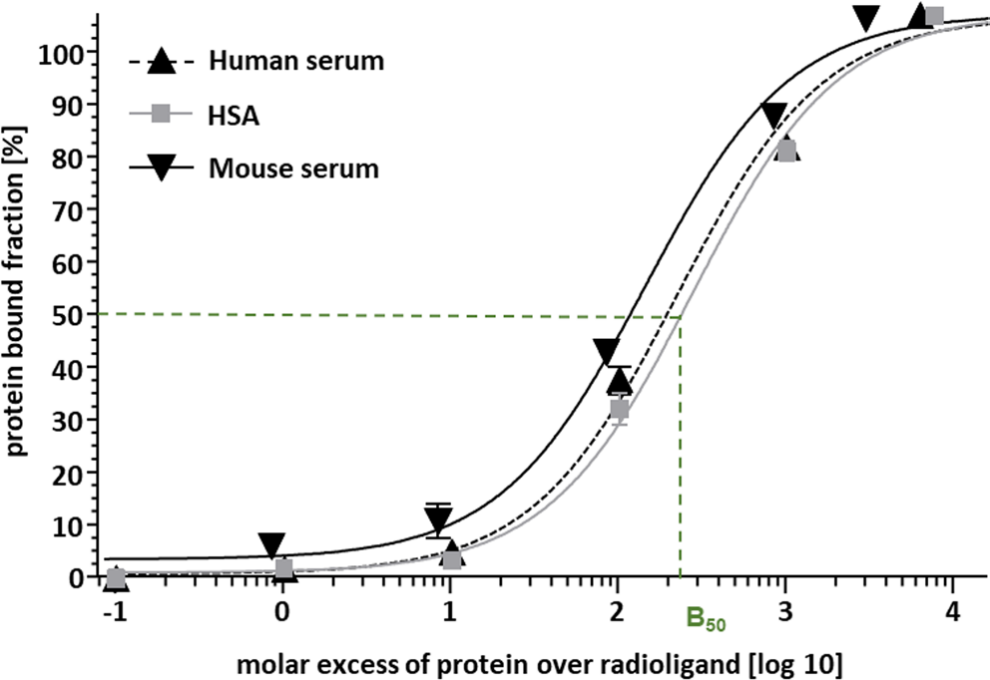
Binding of [^99m^Tc]Tc-PSMA-HSG to human serum, human serum albumin (HSA) and mouse serum as a function of the molar ratio of protein to radioligand (10 pmol). Data are means ± SD from three separate determinations. B_50_ values were calculated via non-linear regression (sigmoidal response) using GraphPad Prism

Of note, a lower B_50_ value indicates that a lower molar excess of protein over radioligand is required to achieve half-maximal protein binding and thus corresponds to a higher plasma protein binding affinity (Fig. 3). Between all compounds investigated, there was a general trend towards higher affinity to plasma proteins (B_50_) with increasing lipophilicity. However, there were also clear differences between compounds in terms of B_50_ for human versus mouse plasma proteins that were not related to logD. For example, while the reference [^99m^Tc]Tc-PSMA-I&S binds twice as strongly to human than mouse plasma proteins, the inverse is the case for [^99m^Tc]Tc-PSMA-HSG and [^99m^Tc]Tc-PSMA-HSG-Cy7-I. [^99m^Tc]Tc-PSMA-HSG-Cy7-II showed a particularly high selectivity for human over mouse protein binding. Generally, there was good overlap between tracer binding to human serum and HSA in PBS, respectively, indicating that HSA is the most relevant contributor to tracer plasma protein binding in human serum.

Overall, in comparison to the reference [^99m^Tc]Tc-PSMA-I&S, [^99m^Tc]Tc-PSMA-HSG showed the most favorable logD and plasma protein binding characteristics. Based on this, its comparably high PSMA-affinity and robust internalization, [^99m^Tc]Tc-PSMA-HSG was selected as the lead candidate for further preclinical evaluation.

### Photophysical characterization of PSMA-HSG

It has been shown previously that sulfonates as substituents on cyanine dyes not only improve solubility and reduce dye stacking, but also positively influence the fluorescent quantum yield [42, 50]. PSMA-HSG showed a molar extinction coefficient of 161.600 L·mol⁻¹cm⁻¹ and a quantum yield of 26% in a 5% human serum albumin solution, yielding a brightness of 42.020 L·mol⁻¹cm⁻¹. These values are in accordance with the values determined for the free dye [51] and lie in the same range as observed for other cyanine-conjugated PSMA-ligands [52].

### In vivo stability study in mice

In a next step, the in vivo stability of [^99m^Tc]Tc-PSMA-HSG in mice at 60 min p.i. was determined via radio-HPLC analysis of blood and urine samples. Due to the low liver uptake (Table 3) and low extraction rates of [^99m^Tc]Tc-PSMA-HSG from mouse liver, the stability of [^99m^Tc]Tc-PSMA-HSG towards hepatic metabolism was assessed in mouse liver homogenates (30 min incubation). After 60 min, the activity in mouse urine was composed entirely of intact [^99m^Tc]Tc-PSMA-HSG. Similarly, in blood samples (60 min p.i.) and liver homogenates (30 min of incubation), the tracer also remained mostly intact, with only minor formation of more hydrophilic metabolites observed (Supplementary Fig. 4).

### In vivo biodistribution and small animal SPECT/fluorescence imaging

To assess the influence of dye conjugation on the in vivo profile of [^99m^Tc]Tc-PSMA-HSG, a comparative biodistribution study, including the reference [^99m^Tc]Tc-PSMA-I&S, was performed in LNCaP xenograft bearing NSG mice. Tracer biodistribution was assessed at 6 h p.i. using a ligand dose of 0.1 nmol/animal [12, 25]. These settings were specifically chosen to reflect the timeline and dosing applied in the clinical situation (surgery at later time points, microdosing: 100 µg/patient). Of note, a ligand amount of 0.1 nmol/mouse (extrapolation: 280 nmol/70 kg patient, amounting to 587 µg/patient for PSMA-HSG) still exceeds the micro-dose (100 µg/patient). Nevertheless, it represents a dose level at which significant competition effects between unlabeled precursor and radioligand for PSMA binding sites in tumor tissue are not probable [53–57]. Additionally, to ensure comparability with literature data on other PSMA-targeted hybrid ligands [52], the biodistribution of [^99m^Tc]Tc-PSMA-HSG was also investigated at 2 h p.i. using a ligand dose of 1 nmol/animal.

**Table 3:** Comparative biodistribution of [^99m^Tc]Tc-PSMA-HSG and [^99m^Tc]Tc-PSMA-I&S (6 h p.i., 0.1 nmol peptide) in LNCaP xenograft bearing male NSG mice (*n* = 5 per group). Biodistribution of [^99m^Tc]Tc-PSMA-HSG was additionally assessed at 2 h p.i. at a peptide dose of 1 nmol (*n* = 5). Data are given in %iD/g and are means ± SD

organ	[^99m^Tc]Tc-PSMA-HSG 6 h, 0.1 nmol	[^99m^Tc]Tc-PSMA-I&S 6 h, 0.1 nmol	[^99m^Tc]Tc-PSMA-HSG 2 h, 1 nmol
blood	1.3 ± 0.3**	0.15 ± 0.03	2.4 ± 0.3**
heart	0.7 ± 0.2**	0.24 ± 0.05	1.3 ± 0.2**
lung	2.5 ± 0.5	2.2 ± 0.2	2.8 ± 0.3
liver	2.5 ± 0.4**	0.8 ± 0.4	3.3 ± 0.3*
spleen	9.7 ± 3.8*	14.2 ± 2.0	5.2 ± 1.9*
pancreas	0.8 ± 0.3	0.7 ± 0.1	0.8 ± 0.1
stomach	1.1 ± 0.2**	0.3 ± 0.1	1.6 ± 0.1**
intestines	1.3 ± 0.2	1.8 ± 0.8	1.0 ± 0.1*
kidneys	148.1 ± 17	147.0 ± 10	61.7 ± 7.2**
salivary glands	4.0 ± 1.2	3.2 ± 0.8	4.2 ± 0.5
muscle	0.4 ± 0.06**	0.2 ± 0.04	0.5 ± 0.04**
LNCaP tumor	19.8 ± 6.6*	12.9 ± 1.4	12.1 ± 0.9*

** *P* < 0.005, * *P* < 0.08 compared to [^99m^Tc]Tc-PSMA-I&S

** *P* < 0.005, * *P* < 0.08 compared to [^99m^Tc]Tc-PSMA-HSG at 2 h p.i.

**Fig. 4:**
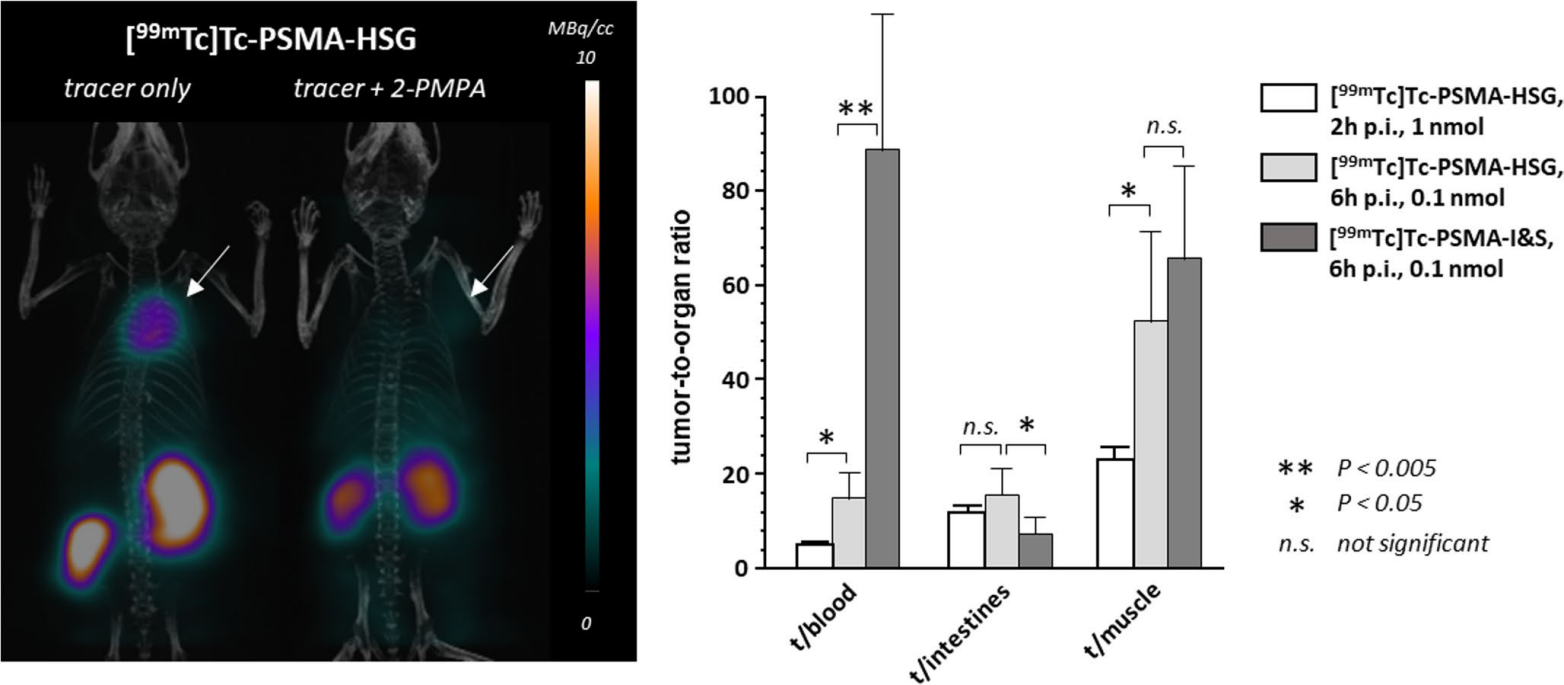
**Left panel**: small animal [^99m^Tc]Tc-PSMA-HSG SPECT/CT of LNCaP xenograft bearing NSG mice 2–4 h p.i. without and with coinjection of 2-PMPA (peptide dose: 1 nmol; blocking dose: 226 µg PMPA = 1 µmol) **Right panel**: Tumor-to-organ ratios of [^99m^Tc]Tc-PSMA-HSG and [^99 m^Tc]Tc-PSMA-I&S for organs of interest in the surgical setting. Data are means ± SD (n = 4–5 animals).

Despite the differences in dosing between the 2 h (1 nmol) and the 6 h (0.1 nmol) time point, [^99m^Tc]Tc-PSMA-HSG showed conclusive clearance and PSMA-targeting kinetics during the entire observation period (Table 3). While background tissue uptake decreased in proportion to blood activity concentration, accumulation in the LNCaP tumor increased by 50% over time, leading to substantially enhanced tumor/background ratios at the later time point (Fig. 4). PSMA-specificity of [^99m^Tc]Tc-PSMA-HSG uptake in the LNCaP xenografts was confirmed via a SPECT/CT imaging study (Fig. 4). Coinjection of a 1000-fold molar excess (1 µmol) of 2-PMPA successfully blocked tumor accumulation of [^99m^Tc]Tc-PSMA-HSG as well as the PSMA-mediated proportion of renal uptake (Fig. 4). Partial blocking of renal activity uptake was also observed in the mice receiving the higher 1 nmol [^99m^Tc]Tc-PSMA-HSG dosing (2 h p.i., Table 3). This well-documented effect [54] led to a near two-fold enhanced the tumor/kidney ratio for [^99m^Tc]Tc-PSMA-HSG at 2 h p.i./1 nmol (0.20 ± 0.03) as compared to 0.12 ± 0.02 at 6 h/0.1 nmol.

The direct side-by-side comparison of [^99m^Tc]Tc-PSMA-HSG and [^99m^Tc]Tc-PSMA-I&S at 6 h p.i. shows strikingly similar overall biodistribution patterns, with the only major difference between the compounds being the substantially slower blood clearance of the hybrid analog as a result of its increased affinity to mouse serum proteins. This leads to overall higher background activity levels at 6 h p.i. (Table 3) and a 50% enhanced tumor accumulation for [^99m^Tc]Tc-PSMA-HSG compared to [^99m^Tc]Tc-PSMA-I&S.

Similar to [^99m^Tc]Tc-PSMA-I&S, high [^99m^Tc]Tc-PSMA-HSG uptake was also observed for the spleen. However, in previous studies, splenic uptake of PSMA-targeted tracers, although partially blockable with 2-PMPA [12, 25], was found to strongly depend on the specific tracer structure and the mouse strain used and not to be mediated by PSMA [25]. Combined, the delayed background clearance of [^99m^Tc]Tc-PSMA-HSG in mice on the one hand and its enhanced tumor accumulation on the other hand lead to similar tumor/intestine and tumor/muscle ratios at 6 h p.i. compared to [^99m^Tc]Tc-PSMA-I&S (Fig. 4).

**Fig. 5:**
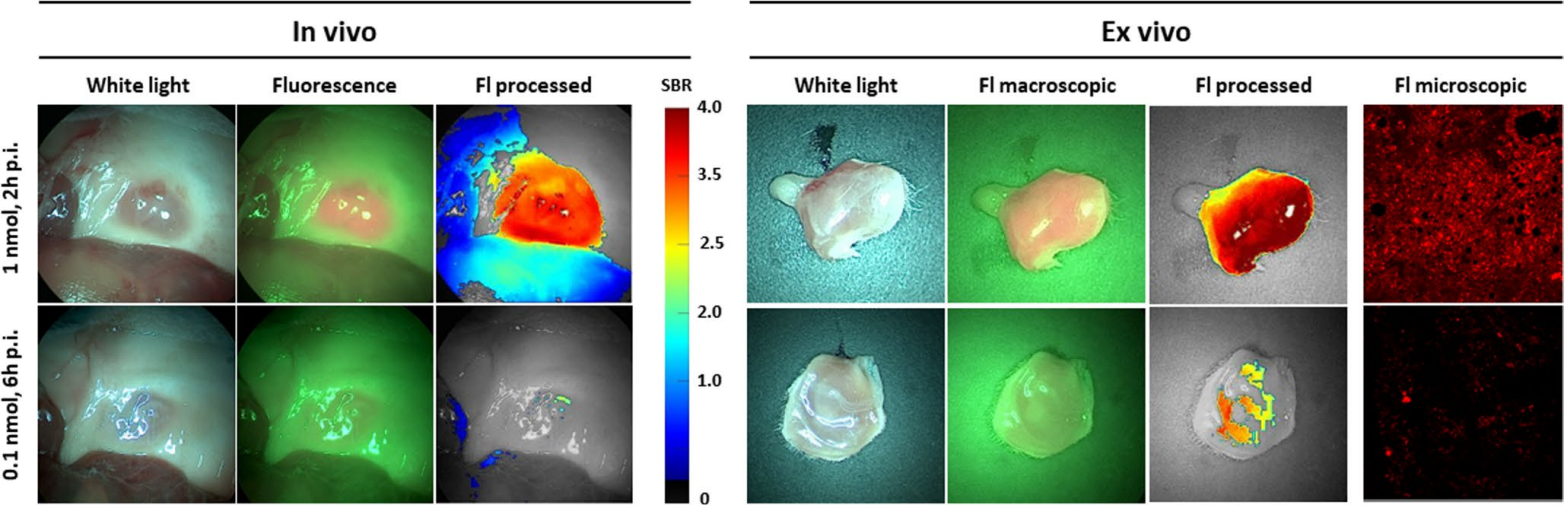
In vivo (**A**) and ex vivo (**B**) fluorescence imaging of PC3-Pip xenografts in NSG mice using PSMA-HSG. Conditions were 2 h p.i. using a peptide dose of 1 nmol/mouse and 6 h p.i. using a peptide dose of 0.1 nmol/mouse, respectively (*n* = 1 per condition). In vivo and ex vivo fluorescence imaging of intact tumors was performed using a clinical-grade fluorescence KARL STORZ Endoscope; fluorescence confocal imaging of 2 mm tumor slices (right) was performed using a Leica SP8 WLL microscope

For the intended application of [^99m^Tc]Tc-PSMA-HSG, i.e. radio- and fluorescence guided surgery of PSMA-overexpressing cancer lesions, high signal-to-background ratios are critical [58, 59]. To validate if the fluorescent intensities and signal-to-background ratios achievable with PSMA-HSG were sufficient to allow fluorescence detection, a fluorescence imaging study was conducted in tumor-bearing mice (Fig. 5). Since LNCaP xenografts tend to be highly hemorrhagic and thus to strongly absorb fluorescent light, PC3-Pip xenograft bearing NSG mice were used for this experiment (Fig. 5). At a ligand dose of 1 nmol (2 h p.i.), high relative PSMA-HSG fluorescence intensity could be detected with a clinical-grade Cy5 fluorescence endoscope [46], while only a weak fluorescence signal was observed at reduced dosing (6 h p.i. using 0.1 nmol ligand). The same applied to confocal fluorescence microscopy of PC3-Pip tumor slices. While the low dose/late timepoint conditions provided a detectable, but weak fluorescence signal, strong and homogenous PSMA-HSG fluorescence was observed under the 2 h p.i./1 nmol conditions. Confocal microscopy revealed that the fluorescent signal was localized both on the cell membrane and inside the tumor cells, indicating progressive ligand internalization.

As documented in the literature, engineered PC3-Pip cells express particularly high PSMA levels, which are not representative for the situation in human tumors (mean number of PSMA binding sites = 4.9 × 10^6^ sites/cell for PC3-Pip cells vs. 5.9 × 10^5^ sites/cell for LNCaP [60]). Thus, to correlate quantitative data with in vivo/ex vivo fluorescence signals, a biodistribution study was conducted under the same conditions as the fluorescence imaging. NSG mice bearing PC3-Pip (PSMA+) and PC3 WT (PSMA-) xenografts on opposite shoulders received [^99m^Tc]Tc-PSMA-HSG (1 nmol), and biodistribution was assessed at 2 h p.i. (Supplementary Table 1). The overall distribution matched previous data from LNCaP-bearing mice (Table 3), but tumor uptake was over twice as high (26.6 ± 5.0%iD/g). Low uptake in PC3 WT tumors (1.6 ± 0.3%iD/g) confirmed PSMA-specificity.

### First-in-man SPECT/CT imaging and dosimetry

#### Patients

Five patients (median age, 64 y; range, 54–78 y) with a diagnosis of initial (*n* = 4) or recurrent (*n* = 1) histologically proven prostate cancer and suspicion on lymph node metastases underwent [^99m^Tc]Tc-PSMA-HSG imaging prior to scheduled radioguided surgery. The median PSA was 8.55 ng/ml (range, 3.7–35) in the patients with newly diagnosed disease, the subject with recurrent PCa presented with a serum biomarker of 2.8 ng/ml. Gleason scores ranged from 7b to 9. Individual patient characteristics are detailed in Supplementary Table 2.

#### Radioligand and patient safety

The administered mass of [^99m^Tc]Tc-PSMA-HSG was 53 µg (25 nmol) in Patients 1–3 (corresponding to the clinical standard dose for [^99m^Tc]Tc-PSMA-I&S imaging and RGS), 350 µg (167 nmol) in Patient 4 and 250 µg (120 nmol) in Patient 5, respectively. The overall injected activity (radiochemical purity > 98%) ranged from 519 to 945 MBq (mean ± SD, 769 ± 164 MBq). Injection of [^99m^Tc]Tc-PSMA-HSG was well tolerated by all five subjects. No side effects or changes in vital signs were observed during the study or follow-up period.

#### Pharmacokinetic analysis and SPECT/CT evaluation

**Fig. 6:**
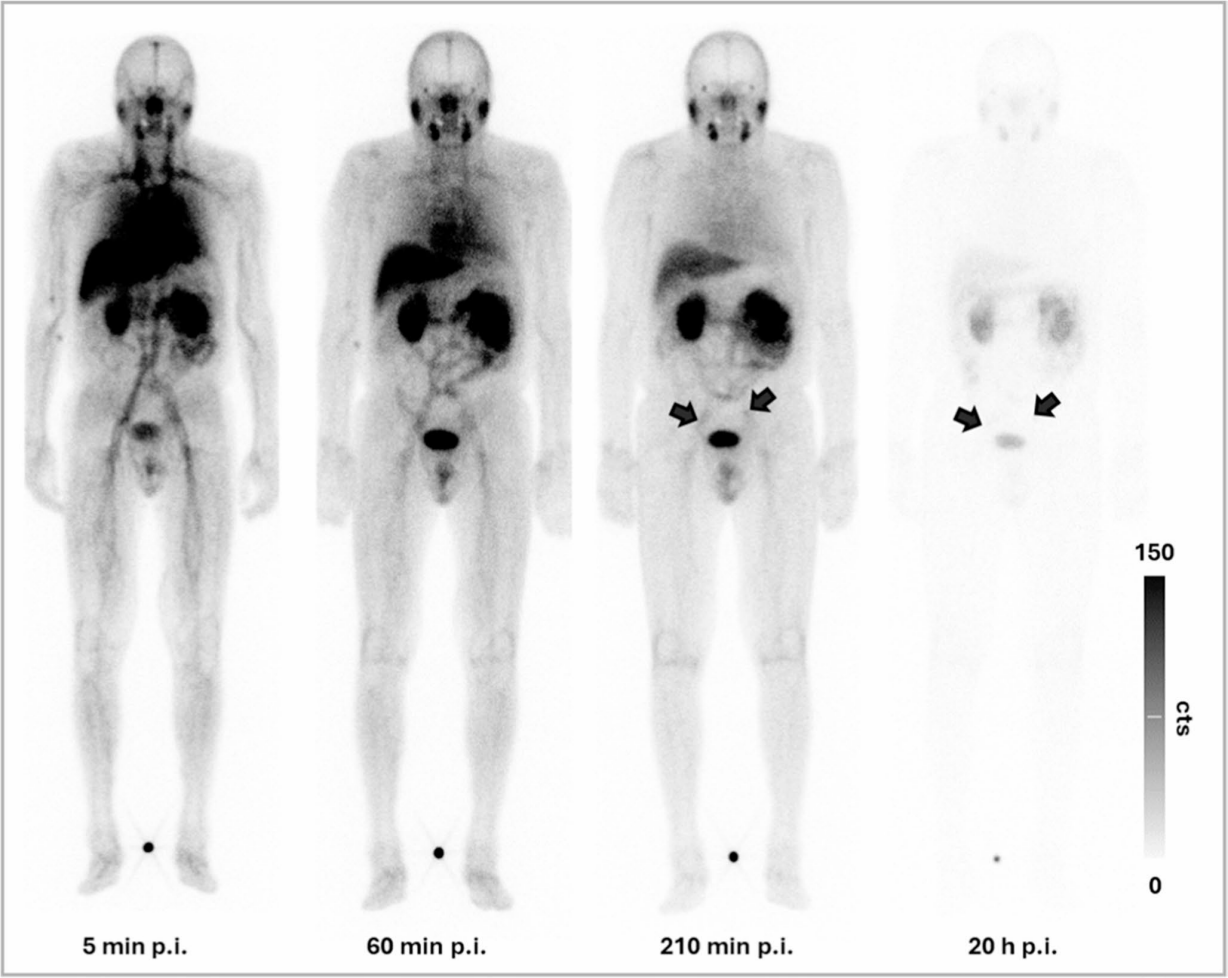
Biodistribution and clearance kinetics of [^99m^Tc]Tc-PSMA-HSG in a patient with a history of prostate carcinoma and biochemical recurrence (patient #1), as determined via sequential planar whole-body scintigraphy (anterior view) (p.i. = post injection)

**Fig. 7:**
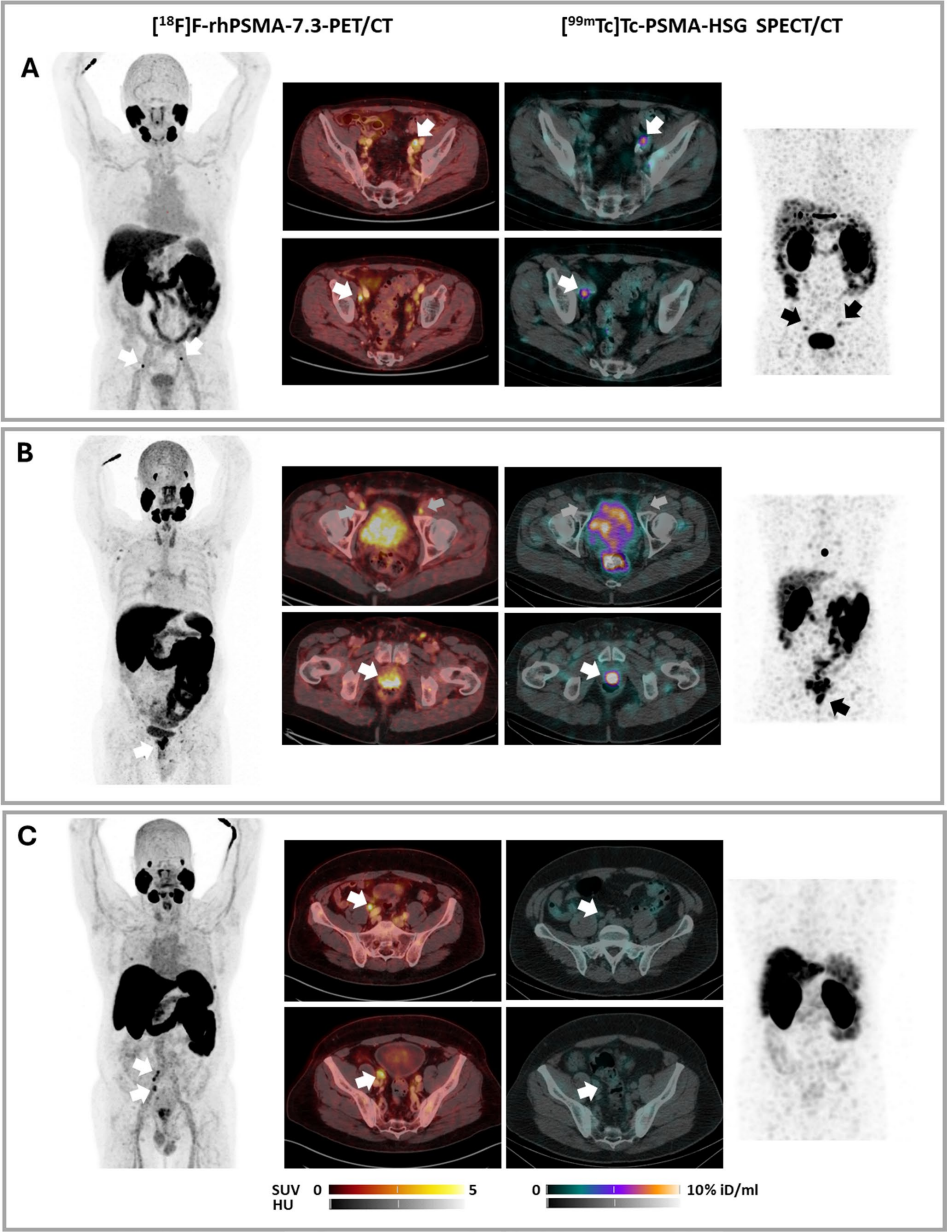
[^18^F]F-rhPSMA-7.3 PET/CT and fused [^99m^Tc]Tc-PSMA-HSG SPECT/CT of Patient #1 (A) Patient #2 (B) and Patient #4 (C). In Patient #1, all PET-positive iliac lymph nodes displayed intense tracer accumulation in [^99m^Tc]Tc-PSMA-HSG SPECT/CT and were confirmed as metastases by histopathology upon RGS. In patient #2, the primary tumor showed intense tracer uptake both in [^18^F]F-rhPSMA-7.3 PET/CT and fused [^99m^Tc]Tc-PSMA-HSG SPECT/CT. Two iliac lymph nodes (grey arrows) with equivocal [^18^F]F-rhPSMA-7.3 uptake were negative in [^99m^Tc]Tc-PSMA-HSG SPECT/CT and were later confirmed histopathologically as true negatives. In Patient #4, PSMA-positive right iliac lymph nodes (*n* = 4) were identified by PSMA-directed PET/CT, but did not show any tracer accumulation in [^99m^Tc]Tc-PSMA-HSG SPECT/CT imaging. However, all four lymph nodes showed tracer accumulation in the ex vivo analysis, and histopathology and ex vivo fluorescence imaging confirmed lymph node metastases and accumulation of PSMA-HSG in all four lymph nodes, consistent with prior PET imaging

Visual image analysis showed swift activity clearance from blood/background via rapid renal excretion in all patients. An example of clearance kinetics and normal biodistribution of [^99m^Tc]Tc-PSMA-HSG is depicted in Fig. 6. In accordance with previous [^18^F]F-rhPSMA-7.3 PET/CT, scintigraphy with [^99m^Tc]Tc-PSMA-HSG allowed successful visualization of two suspicious lymph nodes in the external iliac region in the patient with BCR (Table 4; Fig. 7A). In the subjects with newly diagnosed PCa, the primary tumor could be delineated in 3/4 cases (Fig. 7B). In contrast, only 2/10 lymph nodes that had raised the concern for metastases in PET imaging were positive in [^99m^Tc]Tc-PSMA-HSG scintigraphy. An individual overview of the respective imaging, RGS and subsequent histopathological results is provided in Table 4.

**Table 4:** Findings of [^18^F]F-rhPSMA-7.3-PET/CT, [^99m^Tc]Tc-PSMA-HSG scintigraphy, RGS and histopathologic evaluation in the individual patients

Patient	Indication	[^18^F]F-rhPSMA-7.3-PET/CT	[^99m^Tc]Tc-PSMA-HSG scintigraphy	[^99 m^Tc]Tc-PSMA-HSG RGS	Histopathology
#1	BCR	Two PET-positive LN in the right and left external iliac region, consistent with metastatic disease	Two positive LN in the right and left external iliac region	*In vivo activity measurements*: intraoperative detection feasible; after resection no further tracer accumulation detected *Ex vivo activity measurements*: Right external iliac LN: 230 cps Left external iliac LN: 190 cps no further tracer accumulation	Tumor manifestations in both LN with tracer accumulation in RGS (2/8). Remaining LN (6/8) without tumor infiltration: **pN1 (2/8)**
#2	Initial Staging	PET-positive primary PSMA-positive external iliac LN on both sides	Moderate tracer uptake of the primary No positive LN	*In* and ex vivo activity measurements: No positive LN	Acinar PCa Gleason-score, 5 + 4 = 9, ISUP* 5 No LN metastases: **pN0 (0/12)**
#3	Initial Staging	PET-positive primary Equivocal LN in the left and right external iliac region	Moderate tracer uptake of the primary No positive LN	*In* and ex vivo activity measurements: No positive LN	Acinar PCa Gleason-score 4 + 3 = 7, ISUP 3 No LN metastases: **pN0 (0/10)**
#4	Initial Staging	PET-positive primary PSMA-positive right iliac LN, consistent with metastatic disease	No discernible tracer uptake of the primary No discernible tracer uptake in the right iliac LN	*In vivo activity measurements*: No detection of tracer-positive lesions *Ex vivo activity measurements*: Right iliac LN: 50 cps Right iliac LN: 20 cps (2 adjacent LNs) Right iliac LN: 15 cps	Acinar PCa Gleason-score: 4 + 3 = 7, ISUP 3 Tumor manifestations in all LN with tracer accumulation in RGS (4/4): Remaining LN (16/21) without tumor infiltration: **pN1 (4/21)**
#5	Initial Staging	PET-positive primary Equivocal LN in the left communal iliac region	Moderate tracer uptake of the primary No positive LN	*In* and ex vivo activity measurements: No positive LN	Acinar and cribiform PCa Gleason Score 4 + 3 = 7, ISUP 3 No LN metastases: **pN0 (0/14)**

*ISUP: international society of urological pathology grading system [61]

#### Dosimetry

All patients were included in the dosimetry analysis. Patient 3 had to be excluded from the SPECT-based dosimetry since the reference source activity was not within the imaging FOV.

Supplementary Table 3 lists the SPECT-based organ TIACs for [^99m^Tc]Tc-PSMA-HSG, which were highest for the urinary bladder content (1.904 ± 3.341 h) followed by kidneys (1.870 ± 0.291 h), and liver (1.324 ± 0.888 h). The planar scintigraphy-based TIAC for salivary glands were 0.091 h, 0.043 h, 0.040 h, 0.093 h, and 0.050 h for patients 1 to 5, respectively.

The effective dose coefficient across the four patients with SPECT-based dosimetry was 0.0105 ± 0.0035 mSv/MBq, leading to an effective dose of 7.9 mSv for a typically injected activity of 750 MBq. Highest organ dose coefficients across the four patients with SPECT-based dosimetry were found for kidneys (0.060 ± 0.009 mGy/MBq) followed by urinary bladder wall (0.039 ± 0.075 mGy/MBq), and adrenals (0.024 ± 0.009 mGy/MBq), as summarized in Supplementary Table 4. Absorbed dose coefficient of salivary glands from planar images were 0.007 ± 0.004 mGy/MBq (min: 0.005, max: 0.013) across the five patients.

### Initial clinical experience with [^99m^Tc]Tc-PSMA-HSG RGS

**Fig. 8:**
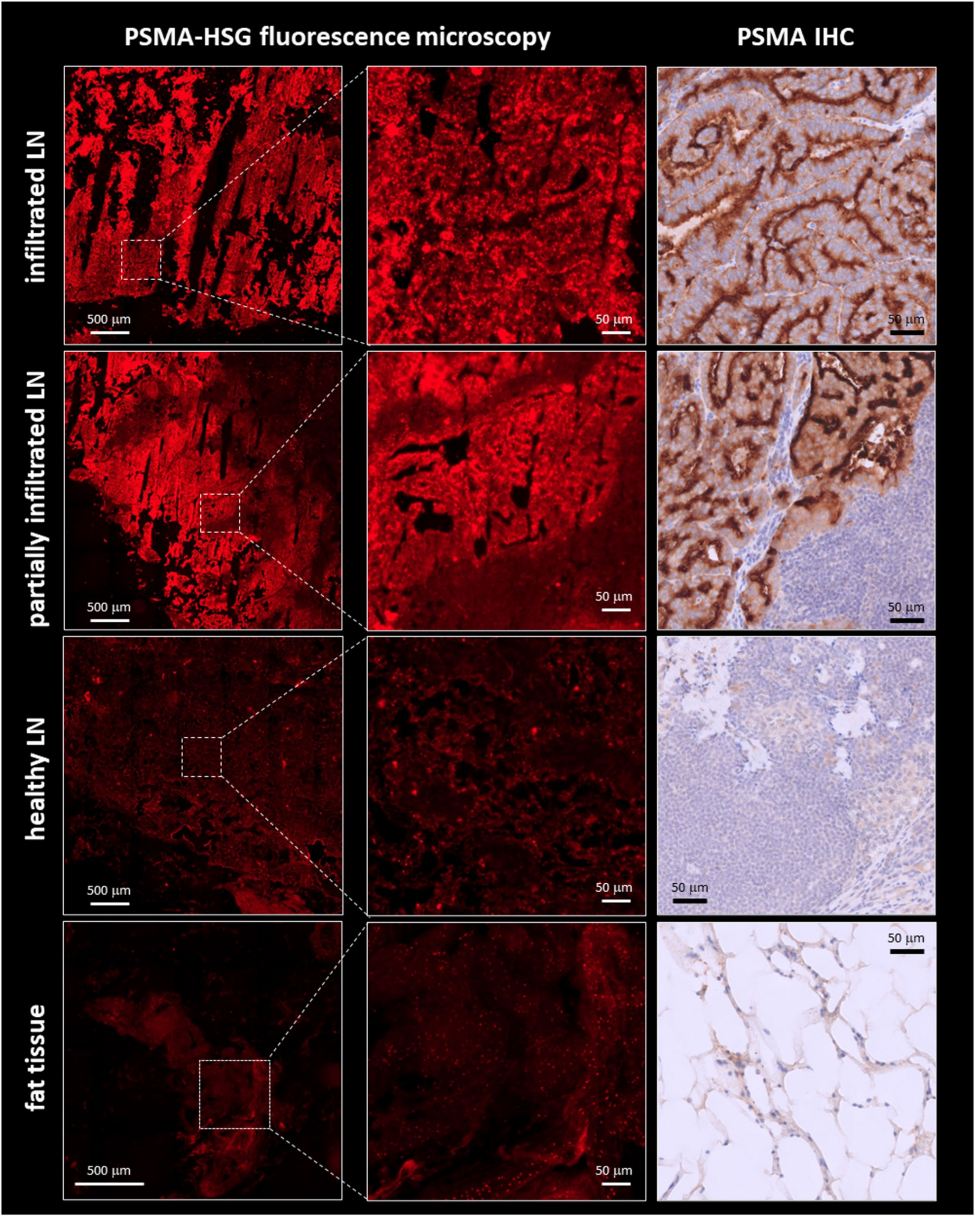
Ex vivo fluorescence microscopy and PSMA-immunohistochemistry of tissue sections (fluorescence: fresh frozen tissue, IHC: paraffinized tissue) of [^99m^Tc]Tc-PSMA-HSG-avid lymph nodes resected during RGS (patient 4) as well as of negative control tissues (healthy lymph node, surrounding fat tissue. Fluorescence microscopy of 6 μm tissue slices was performed using an Axioscan 7 slide scanner (Zeiss)

Overall, in the small patient cohort of this exploratory study, relevant surgical parameters such as signal to background ratios were found not to differ significantly from the experiences gained with [^99m^Tc]Tc-PSMA-I&S. In the patient with BCR (patient #1, Figs. 6 and 7A; Table 4), both suspicious lymph nodes in the external iliac region could be successfully detected in vivo during RGS. Histopathological work-up confirmed the presence of two nodal metastases of PCa (2/8 resected lymph nodes). In the three subjects with newly diagnosed disease (patients #2 (Fig. 7B), #3 and #5), RGS failed to demonstrate any elevated nodal tracer uptake in vivo as well as ex vivo, consistent with absence of lymph node metastases in histopathology (0/36 lymph nodes). In patient #4, who received the highest peptide dose to ensure exemplary fluorescence detection ex vivo [47], four lesions with elevated uptake in PSMA-directed PET were visually not detectable in [^99m^Tc]Tc-PSMA-HSG SPECT (Fig. 7C), but could be successfully identified as tracer-avid lesions during RGS. All four RGS-resected lymphonodal metastases were confirmed histopathologically, and [^99m^Tc]Tc-PSMA-HSG uptake was found to be highly PSMA-specific, as demonstrated by fluorescence microscopy (Fig. 8). Of note, to explore if fluorescence detection would even be possible at a slightly lower dose, patient #5 was injected with 250 µg (120 nmol) of [^99m^Tc]Tc-PSMA-HSG. However, due to non-positivity of all lymph nodes during RGS, no tissues samples were preserved for subsequent fluorescence microscopy, and although highly tracer-positive, the primary tumor was not preserved for subsequent ex vivo analysis.

In summary, a total of 4/4 true positive and 53/53 true negative lymph nodes were identified by means of intra-operative [^99m^Tc]Tc-PSMA-HSG RGS in this patient sub-cohort.

## Discussion

In this translational work we present a unique new tracer design that fulfils the need of high PSMA-specificity and combines the ideal radionuclide characteristics of ^99m^Tc for surgical gamma tracing with a fluorescent signature for target confirmation. This represents the first clinical introduction of a hybrid PSMA tracer that is equivalent or superior to [^99m^Tc]Tc-PSMA-I&S and at the same time can be implemented in the [^99m^Tc]Tc-PSMA-I&S RGS workflow that currently dominates the field of PSMA-targeted RGS [31].

The in vitro evaluation of this novel analog, [^99m^Tc]Tc-PSMA-HSG, revealed superior performance of the SulfoCy5-conjugated agent compared to two alternative SulfoCy7 analogs. This observation is in line with previous findings, demonstrating a similar effect for RGD peptides conjugated with cyanine dyes with different polymethine chain lengths [40]. Furthermore, despite the extension of the linker unit and conjugation with the relatively bulky SulfoCy5 dye (MW = 642 g/mol), [^99m^Tc]Tc-PSMA-HSG outperformed [^99m^Tc]Tc-PSMA-I&S both in vitro (Tables 1 and 2) and in vivo (Table 3). These results are fully in line with the trends observed for the first-generation hybrid ligand [^68^Ga]Ga-PSMA-I&F in comparison to its non-fluorescent parent ligand [^68^Ga]Ga-PSMA-I&T [25].

Despite the fact that the internalization of [^99m^Tc]Tc-PSMA-HSG was substantially decreased compared to [^99m^Tc]Tc-PSMA-I&S (Table 1), both the dual tracer internalization studies (Supplementary Fig. 2) and fluorescence microscopy in vitro (Fig. 2) and ex vivo (Fig. 5) demonstrated that, the hybrid agents did effectively internalize. Interestingly, in other studies, the opposite effect was observed. For distinct series of hybrid PSMA ligands, significant differences in the internalization efficiency (internalized vs. membrane-bound ligand) were found, either as a result of dye structure [27] or of the nature and composition of the linker unit [53, 62]. This suggests that PSMA affinity is only one of multiple parameters contributing to the cellular internalization of PSMA-targeted tracers.

Both lipophilicity (log*D*) and plasma protein binding (PPB) are key physicochemical properties that drive the excretion pathway and general pharmacokinetics of a tracer. For structurally closely related compounds, these two parameters may correlate. However, the extent to which log*D* can represent the interactions of tracers with plasma proteins is highly variable and structure-dependent [63]. The comparative data obtained in this study corroborate this finding (Table 2). The binding to human serum, HSA and mouse serum (B_50_) varied substantially between compounds (see Table 2; Fig. 3). While a correlation between plasma protein binding of the different tracers and their logD was clearly observed for both species (Table 3), the relative affinities for human vs. mouse plasma proteins varied. This needs to be taken into account in the interpretation of comparative in vivo data in mouse models and may influence their translational meaningfulness.

When comparing the comparative in vivo data obtained for [^99m^Tc]Tc-PSMA-HSG vs. [^99m^Tc]Tc-PSMA-I&S (Table 3; Fig. 4), essentially all observed differences seem to be related to the B_50_. The increased affinity of [^99m^Tc]Tc-PSMA-HSG to mouse plasma proteins (Table 2) is reflected in its substantially higher blood activity of (6 h p.i.), which in turn leads to enhanced non-specific background accumulation, e.g. in heart, muscle and liver. The prolonged circulation time, alongside the slightly improved PSMA-affinty of the hybrid tracer, was found to contribute to a 50% increase in tumor uptake of [^99m^Tc]Tc-PSMA-HSG compared to [^99m^Tc]Tc-PSMA-I&S. Since binding of the two compounds to human serum/HSA is similar (see Table 2), however, the enhanced binding of [^99m^Tc]Tc-PSMA-HSG to mouse plasma proteins could have a unique effect on the biodistribution that may not translate to humans.

Indeed, the human biodistribution of [^99m^Tc]Tc-PSMA-HSG (Fig. 6), when compared to literature data on [^99m^Tc]Tc-PSMA-I&S [64], shows faster clearance from the circulation for the hybrid tracer, which is in line with its slightly lower B_50_ for human serum proteins. Furthermore, the same comparison suggests substantially reduced accumulation of [^99m^Tc]Tc-PSMA-HSG in the salivary glands and the spleen. Virtually no intestinal accumulation could be observed for [^99m^Tc]Tc-PSMA-HSG (as opposed to [^99m^Tc]Tc-PSMA-I&S), a feature that may prove advantageous in the surgical setting.

Despite the availability of data indicating that ^99m^Tc is safe to use for RGS [65], one of the common arguments against using radioisotopes for surgical guidance is the use of ionizing radiation. Dosimetry analysis for [^99m^Tc]Tc-PSMA-HSG revealed that there is no such risk. The effective dose for [^99m^Tc]Tc-PSMA-HSG (0.011 ± 0.003 mSv/MBq) was higher compared to [^99m^Tc]Tc-PSMA-I&S (0.0052 mSv/MBq). However, it is substantially lower than the effective doses determined for clinically used PSMA-targeted PET imaging agents such as [^68^Ga]Ga-PSMA-11 (0.023 ± 0.004 mSv/MBq [66]) and [^18^F]DCFPyL (0.0165 mSv/MBq [67]). [^99m^Tc]Tc-PSMA-HSG even displayed reduced kidney absorbed dose coefficients compared to [^99m^Tc]Tc-PSMA-I&S with 0.060 ± 0.009 mGy/MBq and 0.0733 mGy/MBq, respectively [64]. Also, salivary gland absorbed dose coefficients were markedly lower for [^99m^Tc]Tc-PSMA-HSG with 0.007 ± 0.004 mGy/MBq compared to 0.0221 mGy/MBq for [^99m^Tc]Tc-PSMA-I&S. In contrast, the absorbed dose coefficient of the urinary bladder wall was considerably higher with 0.039 ± 0.075 mGy/MBq (vs. 0.000355 mGy/MBq for [^99m^Tc]Tc-PSMA-I&S). However, as different dosimetry workflows were used for the two compounds, it is unclear how significant these differences are.

In terms of lesion detection in patients, [^99m^Tc]Tc-PSMA-HSG SPECT/CT successfully visualized the primary tumor in 3 out of 4 patients, and all lymph node metastases in a single patient. Of note, all lymph nodes that showed equivocal tracer uptake in [^18^F]PSMA PET/CT (patients #2, #3 and #5, see Table 4), were not detectable in preoperative [^99m^Tc]Tc-PSMA-HSG SPECT/CT and were later confirmed to be true negative in subsequent RGS. This finding aligns well with literature data, demonstrating unspecific (false-positive) tracer uptake in preoperative PET observed in particular for lymph nodes at the distal external iliac arteries or outside the pelvis, especially if no PSMA-positive lymph nodes closer to the prostatic fossa were evident [68]. Conversely, all lymph nodes with [^99m^Tc]Tc-PSMA-HSG tracer uptake in RGS were true positive in histopathological work-up, and PSMA-specificity of tracer uptake was additionally corroborated by fluorescence microscopy (Fig. 8). In line with literature, the non-visualization of tracer-avid lesions in [^99m^Tc]Tc-PSMA-HSG SPECT/CT could be compensated for by sensitive identification of tumor-infiltrated lymph nodes during RGS. Thus, in our small exploratory patient cohort, the overall sensitivity, specificity, negative predictive value (NPV) and positive predictive value (PPV) of [^99m^Tc]Tc-PSMA-HSG SPECT/CT was 33%, 100%, 94% and 100% and 100%, 100%, 100% and 100% for RGS. Although larger Phase I/II trials are indispensable for consolidation of these preliminary findings, the obtained results are highly encouraging and so far superior to those obtained for [^99m^Tc]Tc-PSMA-I&S [14, 15]. They also compare favorably to the outcomes achieved with the only other PSMA-targeted hybrid tracer that has been tested in humans so far, namely [^68^Ga]Ga-P3 [30]. At a ligand dose of 40 µg/kg (31 nmol/kg), the sensitivity, specificity, NPV and PPV of [^68^Ga]Ga-P3 PET/CT were 79.1%, 90.4%, 89% and 81.5%, respectively.

Of note, the observed relatively low NPV of [^99m^Tc]Tc-PSMA-HSG SPECT/CT in patient 4 (Fig. 7) may not only be the result of the generally lower sensitivity of [^99m^Tc]Tc-PSMA-HSG SPECT/CT vs. [^18^F]F-rhPSMA-7.3 PET/CT imaging, but may also be related to the relatively high peptide dose (350 µg, 167 nmol) that was administered to patient #4 to achieve sufficient fluorescence signal in the LN metastases. Although a considerably lower amount of compound was administered in the present study compared to other first-in-man studies using PSMA-targeted fluorescent or hybrid ligands [30, 69–71], our data indicates that the excess of “cold” PSMA-HSG over [^99m^Tc]Tc-PSMA-HSG may have led to (partial) competition for binding sites in the PSMA-positive lymph nodes in this patient (Fig. 7C; Table 4). This effect is well documented in preclinical models, with low molar activities consistently leading to reduced tracer uptake, not only in tissues with endogenous PSMA expression (e.g. the kidney, also see Table 3), but also in tumor lesions [54–57, 72]. In humans, no reduction of tumor uptake in PSMA-PET has been observed upon increasing the amount of injected compound tenfold, i.e. by a reduction of the molar activity from 180 to 19 MBq/nmol [73]. Of note, however, the molar activity of [^99m^Tc]Tc-PSMA-HSG administered to patient 4 was 4-fold lower (4.5 MBq/nmol), and thus, an impact on the uptake in the tumor lesions cannot be entirely excluded. This once more illustrates the fundamental trade-off between higher peptide mass (needed for fluorescence detection) and reduced molar activity (risking receptor occupancy and diminished SPECT/RGS signal).

More detailed dose-finding studies in the context of a phase-I/II clinical trial are certainly indispensable to identify the appropriate dose for achieving an optimum signal-to-noise ratio for intraoperative fluorescence guidance [70] without compromising activity uptake for RGS due to receptor saturation. It is encouraging, however, that for [^99m^Tc]Tc-mas_3_-Cy5(SO_3_)-KuE [52], injection of 100 µg (microdose; 71 nmol and hence 1.78 nmol/kg;) in a 40 kg pig allowed fluorescence detection of basal PSMA expression in the pig prostate, using a clinical endoscopic set-up [47]. In patient #4 of this study, the PSMA-HSG dose per kg bodyweight was 3.8 µg/kg (1.8 nmol/kg), which corresponds to the dose used in the above large-animal study. In accordance with dosing studies on the use of ICG-[^99m^Tc]Tc-nanocolloid [74] it may thus be anticipated that micro-dosing could allow combined [^99m^Tc]Tc-PSMA-HSG radio- and fluorescence guided surgery in the future.

## Conclusions

This study presents the successful development, preclinical evaluation, and clinical translation of [^99m^Tc]Tc-PSMA-HSG, a novel PSMA-targeted hybrid tracer designed for radio- and fluorescence-guided surgery (RGS/FGS). Through systematic preclinical in vitro and in vivo structure-activity relationship (SAR) analysis, PSMA-HSG was identified as the optimal candidate within a series of analogs that incorporated different fluorophores and molecular modifications. Translation to a clinical feasibility study indicates that [^99m^Tc]Tc-PSMA-HSG is well tolerated at doses ranging from 53 to 350 µg, with no adverse events reported. The tracer enabled the successful detection and RGS resection of lymph node metastases in all patients with confirmed PSMA-positive disease. Taken together, these findings demonstrate the translational promise of [^99m^Tc]Tc-PSMA-HSG as a next-generation hybrid tracer for precision surgery in prostate cancer. The dual-modality readout, optimized pharmacokinetics, and favorable safety profile position this tracer as a strong candidate for broader clinical implementation. Importantly, the clinical translatability of such hybrid tracers will also benefit from parallel advances in GMP manufacturing, regulatory pathways for dual-label compounds, surgeon and nuclear physician training, and investment in compatible fluorescence imaging systems—considerations that, if addressed collectively, will accelerate translation and maximize clinical impact.

## Supplementary Information

Below is the link to the electronic supplementary material.

Supplementary Material 1

## Data Availability

All data supporting the findings of this study are available within the paper and its Supplementary Information.
