# Supplementary material for: [99mTc]Tc-PSMA-HSG for PSMA-targeted Hybrid Surgical Guidance: a new addition to the PSMA-I&S/I&T family
Source: Eur J Nucl Med Mol Imaging. 2025 Nov 14;53(5):2974–93. doi: 10.1007/s00259-025-07623-2 (PMC13013135; doi:10.1007/s00259-025-07623-2)
Supplement: Supplementary file 1 — Supplementary Material 1 [file 259_2025_7623_MOESM1_ESM.docx]

**Supplementary Material**

**[^99m^Tc]Tc-PSMA-HSG for PSMA-targeted *H*ybrid *S*urgical *G*uidance: a new addition to the PSMA-I&S/I&T family**

Margret Schottelius^1,2,3^*, David Viertl^1^, Tessa Buckle^4^, Hélène Koch^1,2,3^, Sebastian Martin^1,2,3^, Alexandra Litvinenko^1,2,3^, Marianne Patt^5^, D.M. van Willigen^4^, Fijs W.B. van Leeuwen^4^, Hans-Jürgen Wester^6^, Dorothea Weckermann^7^, Alessandro Liebich^5^, Alexander Gäble^5^, Nic G. Reitsam^8,9^, Bruno Märkl^8,9^, Johanna S. Enke^5,9^, Julia Brosch-Lenz^5,10^, Constantin Lapa^5,9^

^1^ Translational Radiopharmaceutical Sciences, Department of Nuclear Medicine and Department of Oncology, Centre Hospitalier Universitaire Vaudois (CHUV) and University of Lausanne, 1011 Lausanne, Switzerland

^2^ AGORA, Pôle de recherche sur le cancer, 1011 Lausanne, Switzerland

^3^ SCCL Swiss Cancer Center Leman, 1011 Lausanne, Switzerland

^4^ Interventional Molecular Imaging Laboratory, Leiden University Medical Center, 2333 ZA Leiden, The Netherlands

^5^ Nuclear Medicine, University of Augsburg, 86156 Augsburg, Germany

^6^ Chair for Pharmaceutical Radiochemistry, Faculties of Chemistry and Medicine, Technische Universität München, 85748 Garching, Germany

^7^ Urology, Medical Faculty, University of Augsburg, 86156 Augsburg, Germany

^8^ Pathology, Medical Faculty, University of Augsburg, 86156 Augsburg, Germany

^9^ Bavarian Center for Cancer Research, Augsburg, 86156 Augsburg, Germany

^10^ Institute of Nuclear Medicine, Glen Burnie, MD, USA

**Peptide Synthesis**

***General information***

Fmoc-(9-fluorenylmethoxycarbonyl-) and all other protected amino acid analogues as well as coupling reagents were purchased from Iris Biotech (Marktredwitz, Germany) or Bachem (Bubendorf, Switzerland). 2-Clorotritylchloride polystyrene (2-CTC) resin was obtained from Iris Biotech, Fmoc-Glu(OtBu)-loaded Wang resin was purchased from Novabiochem/Merck (Darmstadt, Germany). Solvents and all other organic reagents were obtained from Sigma-Aldrich (Munich, Germany), VWR (Dietikon, Switzerland) or Actu-All (Oss, The Netherlands). Commercially available SulfoCy5 and SulfoCy7 (termed SulfoCy7-I throughout the manuscript) free acids were purchased from Lumiprobe (Hannover, Germany). SulfoCy7-II had been prepared previously in our lab in analogy to an established protocol [1]. Solid phase peptide synthesis was carried out manually using an LP-180A syringe shaker (Kamush, Gdansk, Poland).

Analytical reversed-phase high performance liquid chromatography (RP-HPLC) was performed on a MultoKrom 100 C18 (5 μm, 125 × 4.0 mm) column (CS GmbH, Langerwehe, Germany), using a *Shimadzu Corp.* (Kyoto, Japan) system with a LC-40D gradient pump, a CBM-40 system controller, an CTO-40C column oven and a SPD-M40 UV/VIS photodiode array detector. For semi-preparative HPLC, a MultoKrom 100 RP 18 column (5 μm, 150 *x* 10 mm, *CS Chromatographie* *GmbH*, Langerwehe, Germany) with 5 mL/min flow rate was used. Data analysis was performed using the *Shimadzu* Lab Solutions software. Peptides were eluted applying different gradients of 0.1% (v/v) trifluoroacetic acid (TFA) in H_2_O (solvent A) and 0.1% TFA (v/v) in acetonitrile (solvent B) at a constant flow of 1 mL/min (or 5 mL/min for semi-preparative HPLC); specific gradients are cited in the text. Retention times t_R_ are cited in the text. Electrospray ionization mass spectrometry (ESI-MS) was performed using an Advion expression CMS mass spectrometer (Advion, Harlow, UK).

***Synthesis of PSMA-HSG. PSMA-HSG-Cy7-I, PSMA-HSG-Cy7-II and Aad-PSMA-HSG***

*Synthesis of building blocks*

Ac-mercaptoacetyl-D-Ser(tBu)-D-Ser(tBu)-D-Ser(tBu)-OH (Ac-mas_3_(tBu)_3_-OH)

The peptide sequence Fmoc-D-Ser(tBu)-D-Ser(tBu)-D-Ser(tBu)- was assembled manually on 2-CTC resin according to a standard Fmoc SPPS protocol using HOBt and TBTU as coupling reagents. Upon removal of the N-terminal Fmoc protecting group, solid phase coupling with *S*-Acetyl-thioglycolic acid was carried out by adding *S*-Acetyl-thioglycolic acid pentafluorophenyl ester (1.5 eq) and TEA (3 eq) in DMF. After shaking for 2h at RT, the resin was washed with DMF and DCM, and the fully protected mas_3_-chelator was cleaved from the resin using hexafluoroisopropanol in DCM (1:4 (v/v)). The product solution was evaporated to dryness, and upon lyophilization with tBuOH, the crude peptide chelator was obtained in 80% yield based on functional groups on the 2-CTC resin and X% purity (UV-detection at 214 nm).

HPLC (10-90%, 15 min): *t*_R_ = 12.20 min; calculated molecular weight (C_25_H_45_N_3_O_9_S): 563.7; found (ESI-MS): m/z = 564.3 [M+H]^+^

*Fmoc solid-phase peptide synthesis of Ac-mas_3_-k-y-nal-k-Sub-KuE*

Fmoc-Glu(OtBu)-preloaded Wang resin (500 mg, 0.33 mmol) was allowed to preswell in DMF for 30 min. After Fmoc-deprotection using 20% piperidine in DMF and washing with DMF (8x) and DCM (3x), the resin-bound H_2_N-Glu(OtBu) was reacted overnight with 1,1′-Carbonyldiimidazole (1.1 eq), 4-(Dimethylamino)pyridine (0.04 eq) and TEA (2.5 eq) in DMF. After washing with DCM (6x), H-Lys(Fmoc)-OtBu (2 eq) and TEA (3 eq) in DCM were added to the resin, and reaction was allowed to proceed overnight. After washing with DCM (6x) and subsequent swelling in DMF for 30 min, Fmoc-deprotection was carried out using 20% piperidine in DMF. The resin was then washed with DMF (10 x) and reacted overnight with Di-pentafluorophenyl suberate (2 eq)[2] and Diisopropylethylamine (DIPEA, 1.5 eq) in DMF. Upon washing with DMF, the resin was reacted with Fmoc-D-Lys-OtBu (2 eq) and TEA (4 eq) in DMF for 1h and washed with DMF. The subsequent coupling reactions with Fmoc-D-2-Nal-OH, Fmoc-D-Tyr(tBu)-OH and Fmoc-D-Lys(Boc)-OH were carried out according to a standard Fmoc SPPS protocol. For N-terminal functionalization with the fully protected mas_3_-chelator, Ac-mas_3_(tBu)_3_-OH was first preactivated with Pentafluorophenol (1.5 eq), N,N′-Diisopropylcarbodiimide (1.5 eq) and Pyridine (2 eq) in DMF for 2h. Then, the active ester solution was added to the resin that had previously been suspended in DMF containing 1 eq of TEA. After shaking for 2.5 h, the resin was washed with washed with DMF (8x) and DCM (3x), and the inhibitor-peptide construct was cleaved from the resin using TFA containing 2.5% (v/v) Triisopropylsilane. Upon removal of the majority of the solvent *in vacuo*, the crude product was precipitated using Diethyl ether, washed with ether and dried *in vacuo*. Preparative HPLC (20-60% B in 20 min) yielded 10.3 mg of Ac-mas_3_-k-y-nal-k-Sub-KuE in >95% purity.

HPLC (20-60%, 15 min): *t*_R_ = 7.19 min; calculated molecular weight (C_67_H_96_N_12_O_23_S): 1468.6; found (ESI-MS): m/z = 1469.5 [M+H]^+^, 1491.4 [M+Na]^+^, 735.4 [M+2H]^2+^.

*Fmoc solid-phase peptide synthesis of Ac-mas_3_-k-y-nal-k-Sub-Ku-Aad*

For the synthesis of the Aad-analog of PSMA-HSG, dry 2-CTC resin (1 eq of functional groups) was suspended in a solution of Fmoc-Aad(tBu)-OH (2 eq) and DIPEA (4.5 eq) in DCM. After gentle agitation for 2 h, 1 mL MeOH/gram resin was added to cap unreacted functional groups on the resin. After 15 min, the resin was washed with DCM (5x) and with DMF (3x), and the N-terminal Fmoc group was cleaved using 20% piperidine in DMF. All further synthesis steps were identical to the protocol described above for PSMA-HSG.

HPLC (20-60%, 15 min): *t*_R_ = 6.39 min; calculated molecular weight (C_68_H_98_N_12_O_23_S): 1482.6; found (ESI-MS): m/z = 1484.8 [M+H]^+^, 742.6 [M+2H]^2+^.

*Conjugation with fluorescent dyes (free acid)*

The respective peptide backbone (precursors for PSMA-HSG and Aad-PSMA-HSG, respectively; 1 eq) was dissolved in DMF, and 2 eq DIPEA were added. Subsequently, the free carboxylic acid of the respective fluorescent dye (SulfoCy5, Sulfo-Cy7-I, Sulfo-Cy7-II; 1.1 eq) was preactivated with COMU (1.1 eq) and DIPEA (2 eq) in DMF, and the mixture was added to the peptide precursor. Upon completion of the conjugation reaction (after 30-60 min), the crude product was precipitated from diethyl ether, washed and dried in vacuo.

*PSMA-HSG*

The crude product was purified using preparative HPLC (25-40% B in 20 min). PSMA-HSG (Ac-mas_3_-k(Sulfo-Cy5)-y-nal-k-Sub-KuE) was obtained in >97% purity (12% yield based on starting peptide).

HPLC (20-40%, 15 min): *t*_R_ = 10.50 min,

calculated molecular weight (C_99_H_133_N_14_O_30_S_3_): 2095.4; found (ESI-MS): m/z = 1048.3 [M+2H]^2+^.

For quality control RP-HPLC chromatogram and ESI-MS spectrum of the final product please see **Supplementary Figure 1.**

*PSMA-HSG-Cy7-I*

The crude product was purified using preparative HPLC (20-50% B in 20 min). PSMA-HSG-Cy7-I (Ac-mas_3_-k(Sulfo-Cy7-I)-y-nal-k-Sub-KuE) was obtained in >96% purity (21% yield based on starting peptide).

HPLC (20-60%, 15 min): *t*_R_ = 8.95 min; calculated molecular weight (C_104_H_137_N_14_O_30_S_3_): 2159.5; found (ESI-MS): m/z = 1080.7 [M+2H]^2+^, 1091.7 [M+H+Na]^2+^

*PSMA-HSG-Cy7-II*

The crude product was purified using preparative HPLC (20-45% B in 20 min). PSMA-HSG-Cy7-II (Ac-mas_3_-k(Sulfo-Cy7-II)-y-nal-k-Sub-KuE) was obtained in >90% purity (9% yield based on starting peptide).

HPLC (15-45%, 15 min): *t*_R_ = 12.20 min; calculated molecular weight (C_102_H_136_N_14_O_30_S_3_): 2132.9; found (ESI-MS): m/z = 1068.2 [M+2H]^2+^, 1423.4 [2M+3H]^3+^

*Aad-PSMA-HSG*

The crude product was purified using preparative HPLC (25-40% B in 20 min). Aad-PSMA-HSG (Ac-mas_3_-k(Sulfo-Cy5)-y-nal-k-Sub-Ku-Aad) was obtained in >94% purity (12% yield based on starting peptide).

HPLC (20-50%, 15 min): *t*_R_ = 8.57 min; calculated molecular weight (C_100_H_135_N_14_O_30_S_3_): 2109.4; found (ESI-MS): m/z = 1055.1 [M+2H]^2+^.

***Photophysical characterization***

*Molar extinction coefficient*

Of the free dye HOOC-(SO_3_)Cy5(SO_3_)-Methyl (Sulfo-Cy5), a 100 µM stock solution in PBS was prepared. From this, a dilution series comprising the following concentrations was prepared: 7.5 µM, 5 µM, 2.5 µM, 1 µM, 0.75 µM, 0.5 µM, 0.25 µM. Of these solutions, absorption spectra (n=3 per concentration) were recorded using a Shimadzu UV-1280 spectrometer (Shimadzu, Kyoto, Japan). The concentrations were plotted against the mean of the maximum absorbance per concentration. A trendline was fitted, with the regression coefficient x 10^6^ representing the molar extinction coefficient (R^2^ ≥ 0.9900) in M^-1^.L^-1^.cm^-1^.

*Relative quantum yield*

A solution of PSMA-HSG in PBS was prepared. Additionally, a solution of a reference (Sulfonate-(SO_3_)Cy5(SO_3_)-COOH) with a known quantum yield (22% in PBS) was also prepared. Absorption spectra
were recorded at 607 nm (≤ 0.100 Abs) followed by the emission at the same wavelength. Subsequently, 200 µL of each sample was replaced with fresh PBS and again absorption- and emission values were measured. This was repeated 4x, resulting in 6 datapoints for each sample. The absorption- and emission values were plotted in an X-Y scatter plot and a trendline was fitted. The relative quantum yield in % was calculated using the following formula:

ΦF = $\frac{Quantum yield of reference dye}{Slope of the reference dye scatter plot (y)}*Slope of the sample dye scatter plot (y)$

*Brightness*

The brightness in (M^-1^.L^-1^.cm^-1^) was calculated using the following formula:

$$Brightness =Quantum yield hybrid label*Molar extinction coefficient free dye$$

***Plasma Protein Binding***

The plasma protein binding (half-maximal concentration B_50_) of [^99m^Tc]Tc-PSMA-HSG, the two SulfoCy7-derivatives, [^99m^Tc]Tc-PSMA-I&S and [^177^Lu]Lu-PSMA-I&T as an additional reference was evaluated using a previously published protocol with minor modifications [3]. Briefly, protein solutions (maximum concentration: HSA: 758 μM, human serum (total protein): 1284 μM [4], mouse serum (total protein): 988 μM [5]) were diluted with PBS to yield 10^-4^ to 10^-8^ M solutions. The respective PSMA ligand of interest (10 pmol in 10 μL of PBS) was then added to 100 μL aliquots of these solutions, resulting in defined molar ratios between protein and radioligand, ranging from 1:10 to >1000:1 (at maximum physiological protein concentration), respectively. Samples were briefly vortexed and incubated for 30 min at 37 °C. Subsequently, the samples were transferred to VWR centrifugal filter units (500 μL sample volume, PES membrane, 30 kDa cutoff) containing 300 μL PBS and centrifuged at 14’000 g and 4°C for 30 min. The filter units were then removed from the centrifugal tubes and washed twice with 300 μL PBS to recover the protein fraction. The activity in the filtrate, filter and protein fraction were then quantified using a γ-counter. Since varying non-specific binding to the filter membrane was observed for the compounds in this study, the respective activity in the filter unit was included as free radioligand (since only free radioligand can interact with the membrane material during filtration) into the total activity for the calculation of protein-bound vs free radioligand. The percentages of radioligand bound to the respective proteins are represented as means ± standard deviation (SD) of three independent experiments. Plotting against the respective protein-to-radioligand molar ratios and non-linear regression curve fitting (sigmoidal curve fit, log(inhibitor) vs response) using GraphPad Prism (version 10.1.2, Boston, USA) provided the half-maximum binding (B_50_).

***Radiolabeling***

For both PSMA-I&S and PSMA-HSG labeling, kit-like reaction vials containing 5, 10 or 25 nmol of labeling precursor as well as fixed weight proportions of phosphate buffer, sodium tartrate, ascorbic acid, hydrochloric acid and stannous chloride dihydrate (SnCl_2_•2H_2_O) were prepared [2], lyophilized and stored at -20°C until use. For ^99m^Tc-labeling, [^99m^Tc]Tc-pertechnetate (usually 1 GBq) in saline (1.5-2 mL) was added to the sealed reaction vial, which was heated to 95°C for 15 min. Upon cooling, the reaction mixture was diluted with deionized water to a total volume of 10 ml and passed through a SepPak C18 plus (Waters, Eschborn, Germany) cartridge (preconditioned with 5 ml ethanol and 5 ml deionized water). The cartridge was then washed with 6 ml of deionized water and dried with air. For elution, 1 mL of ethanol (0.5% AcOH) was used, and fractions of 3-4 drops of eluate were collected in Eppendorf vials. For animal experiments, the fractions containing the highest amount of activity were combined, evaporated to dryness at 80°C under a nitrogen stream and reconstituted to the required activity concentration for the respective experiment using PBS.

For patient application, where significantly higher amounts of PSMA-HSG were used for radiolabeling (25 -167 nmol), the reaction mixture was directly diluted to a volume of 10 with saline and passed through a sterile filter before i.v. application. Quality control of the final product was performed using Radio-TLC on silica-impregnated glass microfibre chromatography paper (Agilent, Basel, Switzerland), using two different mobile phases: 2-Butanone for determining the amount of free [^99m^Tc]Tc-pertechnetate, and a 1:1 (v/v) mixture of 1M NaOAc/DMF to determine the amount of colloidal [^99m^Tc]Tc-species. For all compounds, the overall radiochemical purity of the final product was always ≥ 98%.

The radioiodinated reference ligand ((^125^I)I-BA)KuE ((S)-1-carboxy-5-(4-(-^125^I-iodo-benzamido)pentyl)-carbamoyl)-L-glutamic acid) was prepared as described [6].

***In vitro evaluation***

*Cell culture*

PSMA overexpressing LNCaP (human prostate carcinoma) cells were grown in DMEM/Nutrition Mix F-12 with Glutamax-I (1:1) (Invitrogen, Life Technologies, Darmstadt, Germany) supplemented with 10% FCS. The prostate carcinoma cell line PC3-PIP was cultured in RPMI-1640 supplemented with 10% heat-inactivated fetal bovine serum, 2 mM L-glutamine, 100 µg/ml penicillin and 100 U/ml streptomycin (Invitrogen, Life technologies). All cell lines were maintained at 37 °C in a humidified 5% CO_2_ atmosphere.

For IC_50_ determination, approximately 150.000 cells/well were seeded on 24-well plates one day prior to the experiment. For internalization studies, 125.000 cells/well in PDL-coated 24-well plates were used. Cell counting was carried out using a Corning cell counter (Cytosmart, Eindhoven, The Netherlands).

*Dual tracer internalization studies*

LNCaP cells were incubated with the respective radioligands (final assay concentration: 0.1 nM for the reference ligand ([^125^I]IBA)KuE, 0.5 nM for the respective ^99m^Tc-labeled compound) for different time points up to 120 min at 37°C. The supernatant was removed, cells were washed with assay medium, and an acid wash (50 mM NaOAc in saline, pH 4.5) was performed to remove membrane-associated activity. Cells were then lysed using 1N NaOH, and internalized activity was quantified using a γ-counter. Data were corrected for non-specific internalization in the presence of 10 µM PMPA (2-(Phosphonomethyl)pentane-1,5-dioic acid; Tocris Bioscience, Bristol, UK) and normalized to the specific internalization observed for the radioiodinated reference compound in the same experiment. Data represent means ± SD (n=3) from three separate determinations.

*Fluorescence microscopy of ligand internalization*

Two days prior to the experiment, LNCaP cells (approximately 100.000 cells/well) were seeded on round glass coverslips placed in the bottoms of the wells of 24-well plates. After cell adhesion and expansion, the culture medium was removed by aspiration, and the cells were washed with HBSS (1% BSA). Then, 200 mL HBSS (1% BSA) and either 25 mL HBSS (1% BSA) (control, n=2 per fluorescent ligand and condition) or 25 μL 1 mM PMPA (blocking, n=2 per fluorescent ligand and condition) were added. Subsequently, 25 μL of PSMA-HSG and Aad-PSMA-HSG (1 μM, final concentration 100 nM) were added, and the cells were either incubated at 4°C for 30 min (membrane binding) or at 60°C for 60 min (internalization). After removal of the supernatant and washing with cold PBS (2x), 250 mL ROTI^®^Histofix (4% Formaldehyde, phosphate buffered, pH 7, Carl Roth AG, Arlesheim, Switzerland) were added, and the cells were incubated for 20 min at RT. The supernatant was then removed, and the cells were washed twice with cold HBSS, followed by the addition of 200 μL DAPI solution (300 nM in HBSS (1%BSA)). After 2 min at RT, the supernatant was removed, the cells were again washed twice with cold PBS, and the coverslips were carefully removed from the 24-well-plate and mounted on glass slides using Invitrogen ProLong^TM^ Antifade Glass mounting medium (Fisher Scientific, Reinach, Switzerland).

Fluorescence images were acquired on a Zeiss Axio Observer Z1 inverted microscope using a Plan Apochromat 20x, N.A. 0.8, air, DIC objective or a Plan Apochromat 63x, N.A. 1.4, oil, DIC objective. Fluorophores used were DAPI and Cy5. Excitation and emission were achieved using a DAPI filter set (excitation BP 365/12, dichroic mirror FT 395, emission LP 397) and Cy5 filter set (excitation BP 650/45, dichroic mirror FT 670, emission BP 710/50). Fluorescence illumination was provided by an X-Cite fluorescence lamp. Images were captured using a Zeiss Axiocam 506 monochrome camera. System operation and image acquisition were performed using the Zeiss Zen 2.6 Blue edition software.

***In vivo evaluation***

*In vivo tumor models*

All in vivo experiments were conducted according to the Swiss Federal Veterinary Office guidelines and were approved by the Cantonal Veterinary Office.

To induce LNCaP tumor growth, a suspension of LNCaP cells (approx. 5 x 10^6^ cells/mouse) in 200 μl of a 1:1 mixture of serum-free DMEM and Matrigel (BD Biosciences) was injected subcutaneously into the right shoulder of male NOD scid gamma (NSG) mice (6-8 weeks, in-house breeding, Dpt. of Oncology, University of Lausanne). After approximately 9 weeks, tumor size reached 4-8 mm in diameter and the mice were used for the in vivo studies.

PC-3 PIP xenografts were established by subcutaneous injection of 5 × 10^6^ PC3-PIP cells/mouse using 8–12-week-old male NSG mice (in-house breeding, Dpt. of Oncology, University of Lausanne) as previously described [7]. Tumors reached a size of 4-8 mm in diameter after 5-6 days and were then immediately used for the fluorescence imaging studies.

*In vivo stability studies*

To determine in the in vivo stability of [^99m^Tc]Tc-PSMA-HSG, 40-50 MBq of tracer in 100 μL PBS were injected intravenously into a male C57BL/6 mouse (n=1). The mouse was placed into a glass beaker, and urine was collected and pooled for 60 min. Subsequently, the animal was sacrificed, and a blood sample (300-500 μL) was collected.

Urine was analyzed via radio-HPLC (Chromolith Performance column, RP-18e, 100x4.6 mm, Merck Millipore, Darmstadt, Germany; 5-60% B in 15 min, 3 mL flow) without further sample preparation. Blood samples were diluted with 1ml of water and centrifuged at 13000 g for 5 min. The supernatant was once more centrifuged at 13000 g for 5 min, and the supernatant of this centrifugation step was then directly analyzed by radio-HPLC.

Due to the poor extraction efficiency of PSMA-targeted ligands from liver, the metabolism of [^99m^Tc]PSMA-HSG by liver enzymes was investigated using a mouse liver homogenate. Liver was homogenized using tissue grinder (2 mL KONTES glass tissue grind pistil) with 1 mL of extraction buffer (1M HEPES, pH 7.4). The homogenate was centrifuged at 13000 g for 5 min. Then, the supernatant was removed and centrifuged once more at 13000 g for 5 min. The resulting clear solution containing liver enzymes was collected, and 200 μl samples were incubated with [^99m^Tc]Tc-PSMA-HSG (10 μl in PBS) at 37°C for 30min and centrifuged at 13000 g for 5 min. The supernatant was then analyzed by radio-HPLC.

*Imaging and Dosimetry Protocol:*

Imaging was performed on a GE Discovery NM/CT 670 Pro (GE Healthcare, Milwaukee, USA; n=4) or Siemens Symbia T2 (Siemens Healthineers, Erlangen, Germany; n=1) SPECT/CT system equipped with a low-energy high-resolution collimator and operating within an energy window of 140.5 keV ± 10%. A scatter window ranging from 114 to 126 keV was additionally acquired for planar and SPECT imaging. For SPECT, the imaging field of view was from neck to upper thighs. At each bed position, 60 views were acquired, with a duration of 8 s each. Subsequently, images were reconstructed using an ordered subset expectation maximisation (OSEM) algorithm (2 iterations and 10 subsets) with a Butterworth filter (f_c_ = 0.48, n = 10). Scatter and attenuation correction was applied.

For whole-body planar imaging, a scan speed of 30 cm/min was employed until 60 min post-injection, after which the acquisition speed was moderated to 12 cm/min for any further data collection. Planar imaging was performed at 5 min, 1 h, 3-4 h and 20 h after injection of the radiopharmaceutical. At 3-4 h and 20 h after injection additional SPECT/CT imaging was acquired.

The planar and SPECT/CT images per time point were processed using syngo.via (Siemens Healthineers, Erlangen, Germany). Organs were segmented on the accompanying CT of the SPECT/CT of each imaging time point to avoid errors due to misalignment during image registration. Segmented organs included gallbladder, muscle, heart, kidneys, liver, lungs, pancreas, spleen, stomach, and urinary bladder on the SPECT/CT. For bone marrow dosimetry, spherical volumes of interest (VOIs) were placed in lumbar vertebrae L2 to L4 [8]. Salivary glands and their background were manually segmented on the anterior view of the planar whole-body scans. Region-wise image statistics were extracted into spreadsheets including administered activity and the time interval between administration and imaging start.

Count statistics were converted into units of Becquerel by using the decay-corrected reference activity source that was within the field of view (FOV) of each imaging acquisition. Percentage injected activities per time were entered into MIRDfit v1.01 for fitting the time-activity data [9]. The trapezoidal method with zero activity at time zero and physical decay extrapolation after the last time point was used for the data with only two SPECT acquisitions and monoexponential fitting was used for the three SPECT acquisitions and if the fit R² was greater than 0.97. The salivary glands with available data from four planar images were fitted biexponentially if R² was greater than 0.97. The bladder was modelled in MIRDfit assuming effective half-life, two time points and a 3 h voiding interval. The resulting time integrated activity coefficients (TIACs, unit of hours) were entered in MIRDcalc v1.22 [10] for dosimetry calculations and patient-individual organ masses for kidneys, liver, pancreas, spleen and estimate for red marrow were used for S-value adjustments.

*Immunohistochemical staining for PSMA:*

Immunohistochemical staining for prostate-specific membrane antigen (PSMA) was performed on formalin-fixed paraffin-embedded (FFPE) tissue sections using the BenchMark Ultra automated staining system (Roche). Antigen retrieval was carried out with Cell Conditioning 1 (CC1) buffer for 32 minutes at 100 °C. Slides were then incubated with a rabbit monoclonal anti-PSMA antibody (clone EP192, BIOO Scientific) at a 1:100 dilution for 36 minutes. Detection was performed using the OptiView DAB IHC Detection Kit (Roche) according to the manufacturer’s protocol.

**Supplementary Tables**

***Supplementary Table 1****: Biodistribution of [^99m^Tc]Tc-PSMA-HSG (2h p.i., 1 nmol peptide) in PC3-Pip xenograft bearing male NSG mice (n=5). Data are given in %iD/g and are means±SD.*

| **organ** | **[^99m^Tc]Tc-PSMA-HSG**  ***2h, 1 nmol*** |
| --- | --- |
| **blood** | 2.8 ± 0.4 |
| **heart** | 1.5 ± 0.3 |
| **lung** | 4.1 ± 1.2 |
| **liver** | 2.9 ± 0.5 |
| **spleen** | 8.2 ± 0.7 |
| **pancreas** | 0.9 ± 0.2 |
| **stomach** | 1.5 ± 0.1 |
| **intestines** | 0.9 ± 0.1 |
| **kidneys** | 100.1 ± 8.2 |
| **salivary glands** | 2.2 ± 0.4 |
| **muscle** | 0.6 ± 0.1 |
| **PC3-Pip tumor** | 26.6 ± 5.0 |
| **PC3 WT tumor** | 1.6 ± 0.3 |

***Supplementary Table 2:*** *Patient characteristics of the five patients in this exploratory study, including initial PSA (iPSA) and injected activity and ligand dose of [^99m^Tc]Tc-PSMA-HSG.*

| **Patient ID** | **Age [y]** | **Height [cm]** | **Weight [kg]** | **Initial Diagnosis** | **iPSA [ng/ml]** | **Gleason**  **Score** | **ISUP**  **Score** | **Histopathology** | **PSA at imaging [ng/ml]** | **Injected Activity [MBq]** | **Ligand dose [μg]** |
| --- | --- | --- | --- | --- | --- | --- | --- | --- | --- | --- | --- |
| **#1** | 76 | 179 | 78 | Aug 22 | 19.6 | 4 + 4 = 8 | 4 | Adenocarcinoma | 1.2 | 769 | 53 |
| **#2** | 58 | 183 | 101 | Apr 24 | 3.6 | 4 + 5 = 9 | 5 | Adenocarcinoma | 4.6 | 519 | 53 |
| **#3** | 73 | 184 | 85 | Jun 24 | 6.0 | 3 + 4 = 7 | 3 | Microacinar Adenocarcinoma | 6 | 741 | 53 |
| **#4** | 55 | 170 | 92 | Jan 25 | 22.0 | 3 + 4 = 7 | 3 | Acinar Adenocarcinoma | 22 | 880 | 350 |
| **#5** | 64 | 170 | 95 | Mar 25 | 13.0 | 4 + 3 = 7 | 3 | Acinar Adenocarcinoma | 13 | 945 | 250 |

***Supplementary Table 3:*** *Time-integrated activity coefficients [h] across organs and patients*.

| **Organ TIAC [h]** | **Patient 1** | **Patient 2** | **Patient 4** | **Patient 5** | **Median** | **SD** |
| --- | --- | --- | --- | --- | --- | --- |
| Gallbladder content | 0.017 | 0.024 | 0.014 | 0.014 | 0.015 | 0.004 |
| Muscle | 1.560 | 1.170 | 1.140 | 1.290 | 1.230 | 0.168 |
| Heart content | 0.169 | 0.159 | 0.211 | 0.141 | 0.164 | 0.026 |
| Kidneys | 2.230 | 1.690 | 1.490 | 2.050 | 1.870 | 0.291 |
| Liver | 1.840 | 2.860 | 0.678 | 0.808 | 1.324 | 0.888 |
| Lungs | 0.764 | 0.391 | 0.328 | 0.305 | 0.360 | 0.190 |
| Pancreas | 0.059 | 0.042 | 0.023 | 0.029 | 0.035 | 0.014 |
| Spleen | 0.098 | 0.275 | 0.052 | 0.093 | 0.096 | 0.087 |
| Stomach content | 0.104 | 0.128 | 0.046 | 0.075 | 0.090 | 0.031 |
| Urinary bladder content | 0.458 | 0.230 | 8.410 | 3.350 | 1.904 | 3.341 |
| Bone marrow - red (active) | 0.734 | 0.478 | 0.251 | 0.346 | 0.412 | 0.182 |

***Supplementary Table 4:*** *Target Organ Absorbed dose [mGy/MBq] across organs and effective dose [mSv/MBq] from SPECT-based dosimetry.*

|  | **Patient 1** | **Patient 2** | **Patient 4** | **Patient 5** | **Median** | **SD** |
| --- | --- | --- | --- | --- | --- | --- |
| Adipose tissue | 0.003 | 0.002 | 0.005 | 0.003 | 0.003 | 0.001 |
| Adrenals | 0.028 | 0.026 | 0.017 | 0.022 | 0.024 | 0.005 |
| Bone - endosteal cells | 0.004 | 0.003 | 0.006 | 0.004 | 0.004 | 0.001 |
| Bone marrow - red (active) | 0.007 | 0.006 | 0.010 | 0.007 | 0.007 | 0.002 |
| Brain | 0.000 | 0.000 | 0.000 | 0.000 | 0.000 | 0.000 |
| Breast tissue | 0.003 | 0.003 | 0.002 | 0.002 | 0.002 | 0.001 |
| Bronchial basal cells | 0.021 | 0.013 | 0.010 | 0.009 | 0.011 | 0.005 |
| Bronchial secretory cells | 0.021 | 0.013 | 0.010 | 0.009 | 0.011 | 0.005 |
| Bronchiolar secretory cells | 0.018 | 0.012 | 0.008 | 0.008 | 0.010 | 0.005 |
| Colon - ICRP133 | 0.006 | 0.006 | 0.015 | 0.009 | 0.008 | 0.004 |
| Colon - left | 0.006 | 0.006 | 0.005 | 0.005 | 0.005 | 0.001 |
| Colon - rectosigmoid | 0.004 | 0.002 | 0.045 | 0.019 | 0.011 | 0.020 |
| Colon - right | 0.008 | 0.008 | 0.007 | 0.007 | 0.007 | 0.001 |
| Esophagus | 0.007 | 0.007 | 0.004 | 0.004 | 0.005 | 0.002 |
| ET1 airway basal cells | 0.000 | 0.000 | 0.000 | 0.000 | 0.000 | 0.000 |
| ET2 airway basal cells | 0.001 | 0.001 | 0.000 | 0.001 | 0.001 | 0.000 |
| Extrathoracic region - ICRP133 | 0.001 | 0.001 | 0.000 | 0.001 | 0.001 | 0.000 |
| Eye lens | 0.000 | 0.000 | 0.000 | 0.000 | 0.000 | 0.000 |
| Gallbladder wall | 0.020 | 0.025 | 0.010 | 0.012 | 0.016 | 0.007 |
| Heart wall | 0.009 | 0.009 | 0.005 | 0.005 | 0.007 | 0.002 |
| Kidneys | 0.051 | 0.063 | 0.056 | 0.071 | 0.060 | 0.009 |
| Liver | 0.028 | 0.032 | 0.013 | 0.015 | 0.021 | 0.009 |
| Lung - ICRP133 | 0.014 | 0.010 | 0.007 | 0.007 | 0.009 | 0.004 |
| Lungs (AI) | 0.014 | 0.010 | 0.007 | 0.007 | 0.009 | 0.004 |
| Lymph nodes - extrathoracic | 0.001 | 0.001 | 0.001 | 0.001 | 0.001 | 0.000 |
| Lymph nodes - systemic | 0.006 | 0.005 | 0.014 | 0.008 | 0.007 | 0.004 |
| Lymph nodes - thoracic | 0.005 | 0.004 | 0.003 | 0.003 | 0.004 | 0.001 |
| Lymphatic nodes - ICRP133 | 0.005 | 0.005 | 0.012 | 0.007 | 0.006 | 0.003 |
| Muscle | 0.003 | 0.002 | 0.005 | 0.003 | 0.003 | 0.001 |
| Oral mucosa | 0.001 | 0.001 | 0.000 | 0.001 | 0.001 | 0.000 |
| Pancreas | 0.019 | 0.020 | 0.011 | 0.013 | 0.016 | 0.004 |
| Pituitary gland | 0.001 | 0.000 | 0.000 | 0.000 | 0.000 | 0.000 |
| Prostate | 0.005 | 0.003 | 0.074 | 0.030 | 0.018 | 0.033 |
| Skin | 0.001 | 0.001 | 0.002 | 0.001 | 0.001 | 0.000 |
| Small intestine | 0.006 | 0.006 | 0.017 | 0.010 | 0.008 | 0.005 |
| Spleen | 0.019 | 0.019 | 0.010 | 0.012 | 0.015 | 0.004 |
| Stomach | 0.010 | 0.012 | 0.006 | 0.007 | 0.009 | 0.003 |
| Testes | 0.001 | 0.001 | 0.005 | 0.002 | 0.002 | 0.002 |
| Thymus | 0.004 | 0.003 | 0.002 | 0.002 | 0.003 | 0.001 |
| Thyroid | 0.003 | 0.002 | 0.001 | 0.001 | 0.002 | 0.001 |
| Tongue | 0.001 | 0.001 | 0.000 | 0.001 | 0.001 | 0.000 |
| Tonsils | 0.001 | 0.001 | 0.001 | 0.001 | 0.001 | 0.000 |
| Ureters | 0.009 | 0.007 | 0.025 | 0.014 | 0.012 | 0.008 |
| Urinary bladder wall | 0.011 | 0.006 | 0.168 | 0.068 | 0.039 | 0.075 |
| **Effective Dose [mSv/MBq]** | **0.010** | **0.009** | **0.017** | **0.011** | **0.011** | **0.003** |

**Supplementary Figures**

**
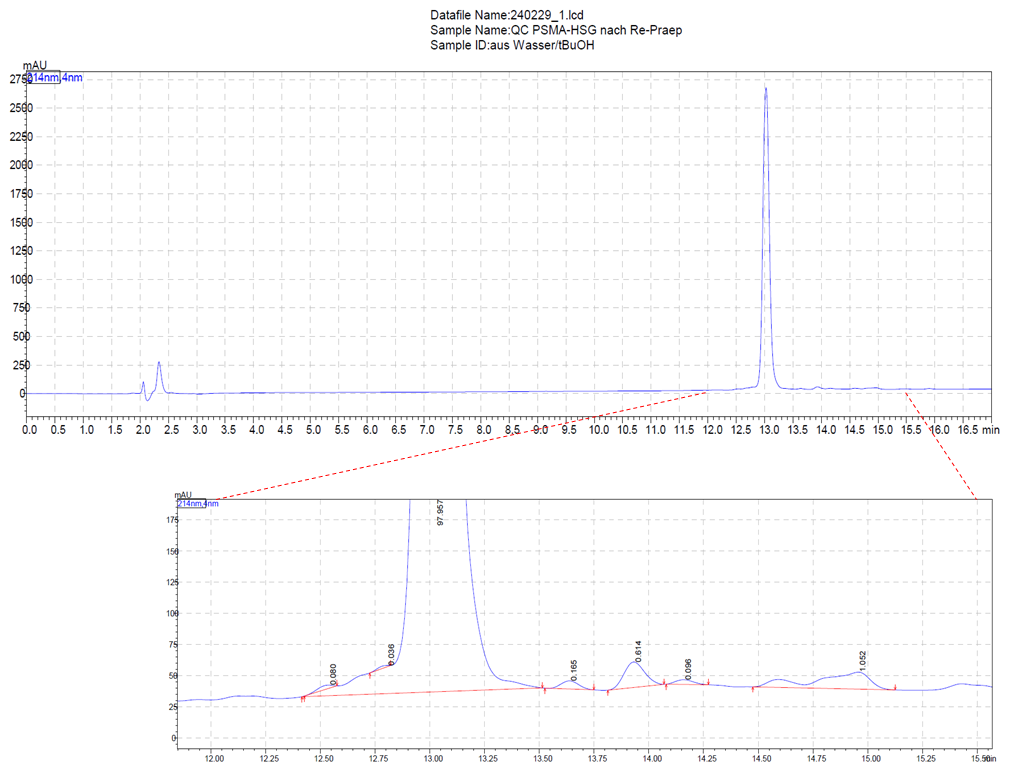
**


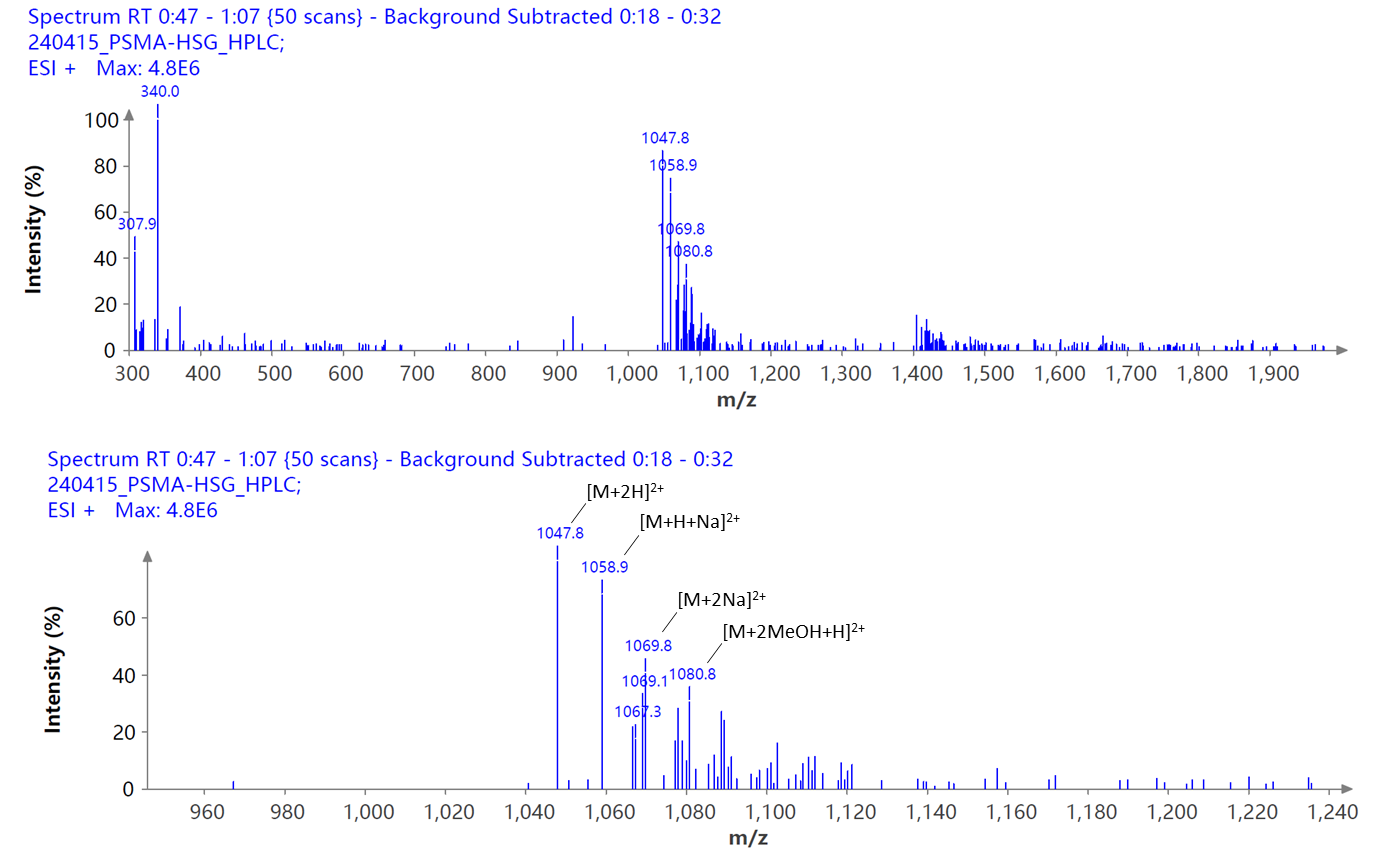


***Supplementary Figure 1****: Upper panel: RP-HPLC quality control of PSMA-HSG. For analytical RP-HPLC, a MultoKrom 100-5 C-18, 125 x 4.6 mm column was used, applying a gradient of 10-40% acetonitrile (0.1% TFA) in water (0.1% TFA) over 15 min at a constant flow rate of 1 mL/min. Lower panel: ESI mass spectrometry characterization of PSMA-HSG.*


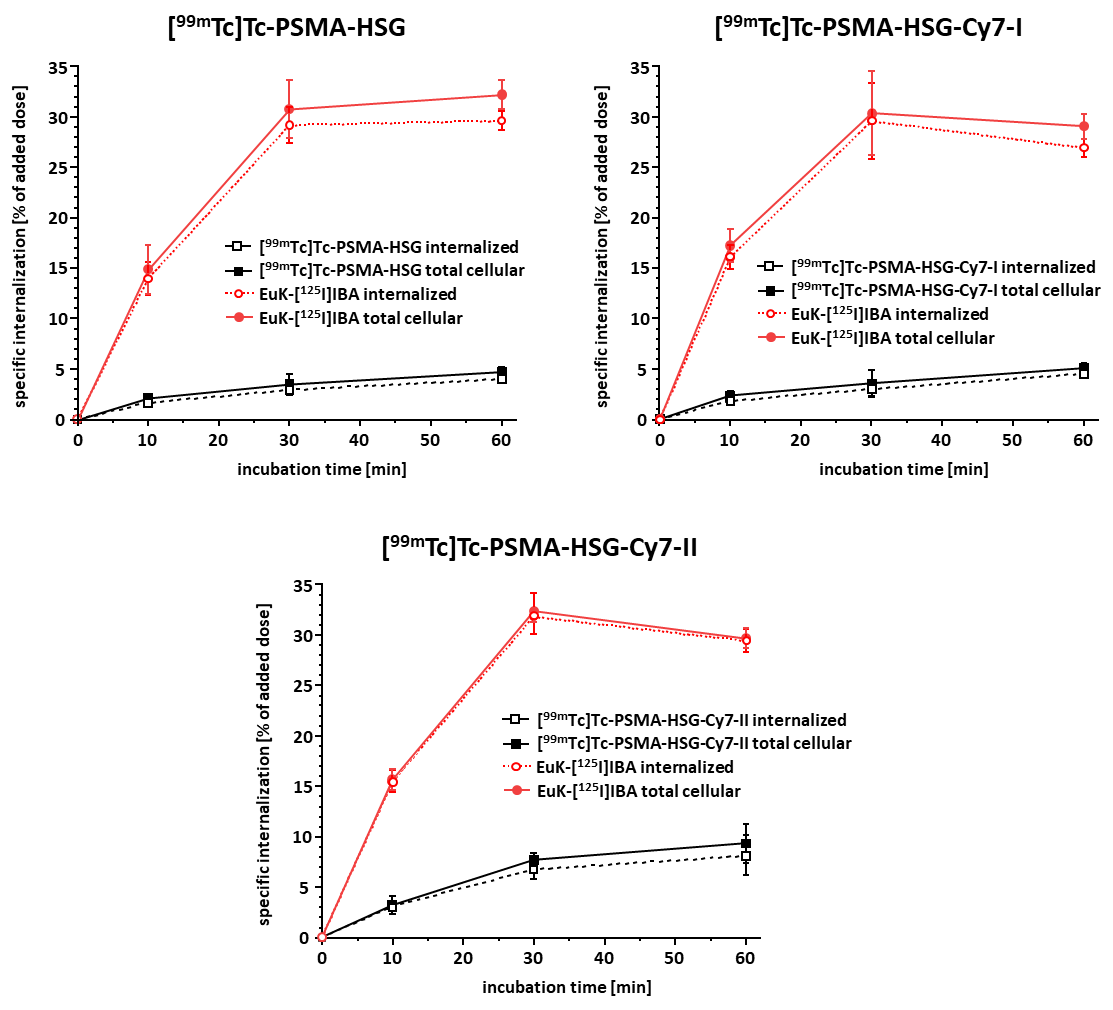


***Supplementary Figure 2****: Internalization kinetics of [^99m^Tc]Tc-PSMA-HSG, [^99m^Tc]Tc-PSMA-HSG-Cy7-I and [^99m^Tc]Tc-PSMA-HSG-Cy7-II (1 nM) in LNCaP cells. Experiments were performed as dual tracer internalization studies, including EuK-[^125^I]IBA (0.1 nM) as an internal reference. Data represent means±SD of n=3 samples and are corrected for non-specific binding/internalization in the presence of 10 μM 2-PMPA. Total cellular activity represents the sum of internalized and membrane bound (acid releasable) activity.*


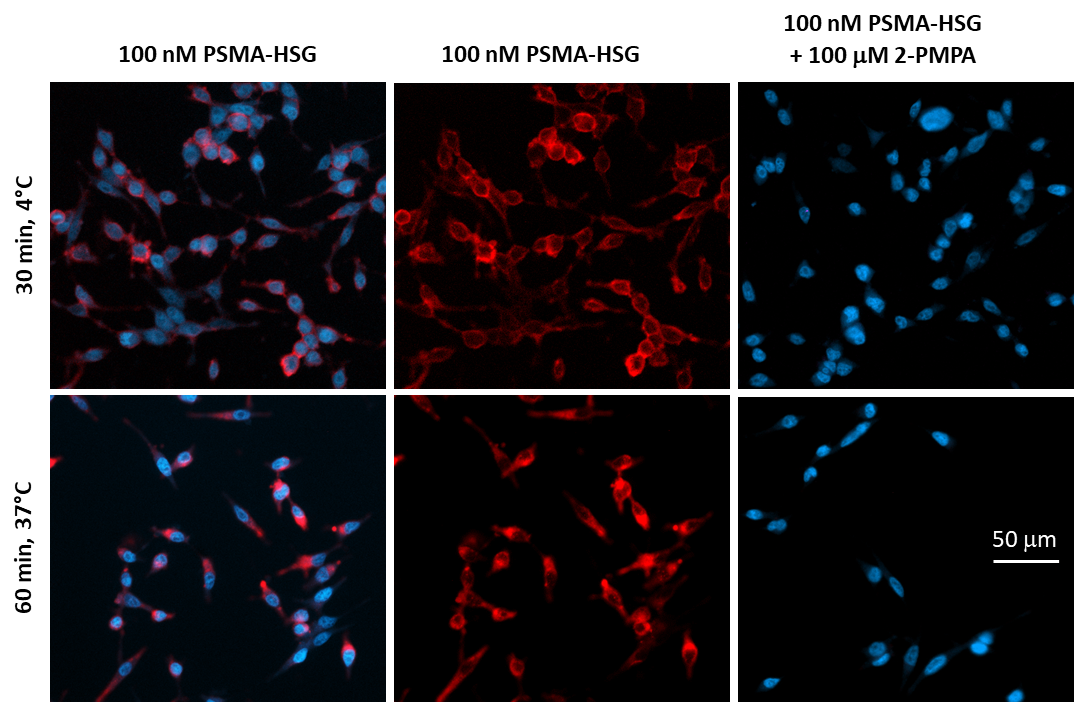


***Supplementary Figure 3****: Fluorescence microscopy (20-fold magnification) of LNCaP cells after incubation with 100 nM PSMA-HSG for 30 min at 4°C (membrane binding, upper row) and for 60 min at 37°C (internalization, lower row). Cells were fixed with 4% paraformaldehyde, and nuclei were stained with 300 nM DAPI. Specificity of PSMA-HSG binding and internalization was demonstrated by coincubation with 100 μM 2-PMPA. Microscope settings and image parameters were kept constant for all samples in the respective groups to allow for visual quantification of Cy5 signal intensity.*


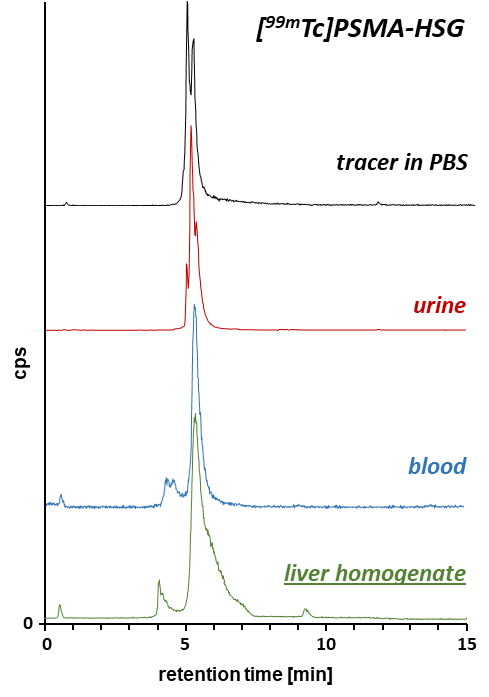


***Supplementary Figure 4****: Radio-RP-HPLC analysis of [^99m^Tc]PSMA-HSG before i.v. injection (black chromatogram) and 1h p.i. in mouse urine (red chromatogram) and mouse blood (blue chromatogram) and after 30 min of incubation at 37° in mouse liver homogenates (green chromatogram). The metabolite analysis were performed in a male Black Six mouse. For radio-RP-HPLC, a Chromolith Performance column, RP-18e, 100x4.6 mm (Merck Millipore, Darmstadt, Germany) was used, applying a gradient of 5-60% B in 15 min and a constant flow rate of 3 mL/min.*

**References**

1. van der Wal S, Kuil J, Valentijn ARPM, van Leeuwen FWB, Synthesis and systematic evaluation of symmetric sulfonated centrally C-C bonded cyanine near-infrared dyes for protein labelling. Dyes and Pigments. 2016; 132*:* 7-19.

2. Robu S, Schottelius M, Eiber M, Maurer T, Gschwend J, Schwaiger M, et al., Preclinical Evaluation and First Patient Application of 99mTc-PSMA-I&S for SPECT Imaging and Radioguided Surgery in Prostate Cancer. J Nucl Med. 2017; 58*:* 235-242.

3. Deberle LM, Tschan VJ, Borgna F, Sozzi-Guo F, Bernhardt P, Schibli R, et al., Albumin-Binding PSMA Radioligands: Impact of Minimal Structural Changes on the Tissue Distribution Profile. Molecules. 2020; 25.

4. Cameron JM, Bruno C, Parachalil DR, Baker MJ, Bonnier F, Butler HJ, et al., Chapter 10 - Vibrational spectroscopic analysis and quantification of proteins in human blood plasma and serum. In *Vibrational Spectroscopy in Protein Research - From Purified Proteins to Aggregates and Assemblies*, Academic Press: 2020; pp 269-314.

5. Zaias J, Mineau M, Cray C, Yoon D, Altman NH, Reference values for serum proteins of common laboratory rodent strains. J Am Assoc Lab Anim Sci. 2009; 48*:* 387-90.

6. Weineisen M, Simecek J, Schottelius M, Schwaiger M, Wester HJ, Synthesis and preclinical evaluation of DOTAGA-conjugated PSMA ligands for functional imaging and endoradiotherapy of prostate cancer. EJNMMI Res. 2014; 4*:* 63.

7. Giordano-Attianese G, Gainza P, Gray-Gaillard E, Cribioli E, Shui S, Kim S, et al., A computationally designed chimeric antigen receptor provides a small-molecule safety switch for T-cell therapy. Nat Biotechnol. 2020; 38*:* 426-432.

8. Hindorf C, Glatting G, Chiesa C, Linden O, Flux G, Committee ED, EANM Dosimetry Committee guidelines for bone marrow and whole-body dosimetry. Eur J Nucl Med Mol Imaging. 2010; 37*:* 1238-50.

9. Carter LM, Ocampo Ramos JC, Schuerrle SB, Marquis H, Lassmann M, Bolch WE, et al., MIRD Pamphlet No. 30: MIRDfit-A Tool for Fitting of Biodistribution Time-Activity Data for Internal Dosimetry. J Nucl Med. 2024; 65*:* 1808-1814.

10. Kesner AL, Carter LM, Ramos JCO, Lafontaine D, Olguin EA, Brown JL, et al., MIRD Pamphlet No. 28, Part 1: MIRDcalc-A Software Tool for Medical Internal Radiation Dosimetry. J Nucl Med. 2023; 64*:* 1117-1124.
